# The role of interpersonal synchrony in forming impressions of autistic and non-autistic adults

**DOI:** 10.1038/s41598-023-42006-3

**Published:** 2023-09-18

**Authors:** I. S. Plank, L. S. Traiger, A. M. Nelson, J. C. Koehler, S. F. Lang, R. Tepest, K. Vogeley, A. L. Georgescu, C. M. Falter-Wagner

**Affiliations:** 1 Department of Psychiatry and Psychotherapy, Medical Faculty, Ludwig-Maximilians-University, Nussbaumstraße 7, 80336 Munich, Germany; 2 Department of Psychiatry and Psychotherapy, Faculty of Medicine and University Hospital Cologne, University of Cologne, Cologne, Germany; 3 Department of Psychology, Institute of Psychiatry, Psychology and Neuroscience, King’s College London, London, UK

**Keywords:** Human behaviour, Medical research

## Abstract

When people meet, they almost instantaneously form an impression of each other. First impressions of character traits and rapport are less favourable when people with autism spectrum condition (ASC) are judged compared to non-autistic people. Little is known about the behavioural differences that drive these altered impressions. In the present study, we investigated the influence of interpersonal synchrony on impression formation of autistic and non-autistic people. Specifically, we used lagged cross-correlations to assess how much each interactant’s motion energy, a measure which can be determined from video recordings, influenced the other interactant’s motion energy. In short, silent clips of dyadic conversations, we asked non-autistic participants to rate their impression of one of the two interactants, which was solely based on the outlines of both interactants. We expected that the amount of leading of the target interactant, their diagnostic status as well as the interaction of these factors would influence impression formation. We found that while the amount of leading had a positive effect on the impressions of non-autistic interactants, this was not true for interactants with ASC. This suggests that interpersonal synchrony of motion energy is one driver of less favourable impressions of autistic compared to non-autistic people.

## Introduction

### Influence of ASC on impression formation

Studies have shown that autistic individuals differ from non-autistic individuals both in terms of verbal and nonverbal communication^1–4^. These differences influence how non-autistic individuals perceive autistic people that they encounter. Indeed, multiple studies show that first impressions of autistic individuals are less favourable^5–9^. First impressions as automatic judgments are quick and based on limited information^10^. Nonetheless, they substantially influence subsequent interactions and judgements^11^ and predict whether friendships are formed^12^. This has a profound impact on the lives of autistic individuals^8,13,14^. Therefore, it is imperative to understand why autistic people, routinely receive a less favourable first impression.

In several studies, Sasson and colleagues demonstrated that these findings regarding first impressions of ASC persist across different modalities^5,8^. First, they investigated how non-autistic participants rated audio sequences, silent videos, videos with tone, static images and transcripts of speech of individuals with and without ASC in a mock audition for a TV show^5^. They found that in all modalities, except transcribed speech content, individuals with ASC were rated less positively than individuals without ASC. This finding suggests that the judgements are not unfavourable due to content but rather the quality of communication; possibly due to differences in pronunciation, intonation, gestures, facial expressions, and other verbal and nonverbal features.

While these effects were robust across stimulus types and experimental settings, the effect of diagnostic status on impression formation was decreased when non-autistic participants were aware of the existing ASC diagnosis of the rated person^8^. This attenuation suggests that in real life, non-autistic people might be more willing to adjust their first impressions if they are aware of a diagnosis. Notably, the adjustment of first impressions seems to depend more on the rater than the rated person^9^. Specifically, a higher stigma against autistic individuals was associated with less favourable ratings. This bias also interacted with the positive effect of awareness of the diagnosis and reversed it such that non-autistic participants who showed greater stigma attribution gave less favourable ratings if they knew about a person’s diagnosis.

Interestingly, the unfavorable first impressions of individuals with ASC is not limited to the observations of non-autistic individuals but also extends to judgements made by other autistic individuals. DeBrabander et al.^7^ showed brief videos of adults with and without ASC to participants with and without ASC. They found a main effect of stimulus diagnosis: autistic individuals were rated less favourably on average compared to non-autistic individuals, which was independent of the diagnostic status of the rater.

### Impression formation and interpersonal synchrony

Despite the importance of impression formation for everyday life and typical social interactions, it is still unclear which mechanisms and behavours specifically lead to individuals with ASC receiving less favourable judgements. Impression formation often focuses on rapport, for example, asking raters to judge someone’s likeability^5,7,9^. Interpersonal synchrony has been used to indirectly but objectively measure rapport^15–19^ and is the phenomenon where people temporally coordinate their behaviour and brain waves when interacting^20,21^. Such coordination helps establish a common ground between interaction partners. Interpersonal synchrony is assumed to help foster relationships and create social bonds^15,16,18,19,22–24^. Vacharkulksemsuk and colleagues^15^ asked strangers to interact with each other either focusing on self-disclosure or on a scientific article. They found that self-disclosure increased interpersonal synchrony which in turn was associated with higher rapport with their interaction partner. Similarly, Lakin and Chartrand^18^ showed that participants unconsciously use more behavioural mimicry, a form of interpersonal synchrony, if their goal is to create rapport with someone. Interestingly, moving synchronously also increases bonding, especially with out-group members^25^. Additionally, we are more likely to synchronise our behaviour with in-group members, even if membership is arbitrary^26^. These results suggest a bidirectional effect: people use synchrony to achieve rapport which in turn increases synchrony. One mediating factor for these effects could be how well we understand our interaction partner’s mental state with a study showing that synchrony can help us understand others^22^.

**Figure 1:**
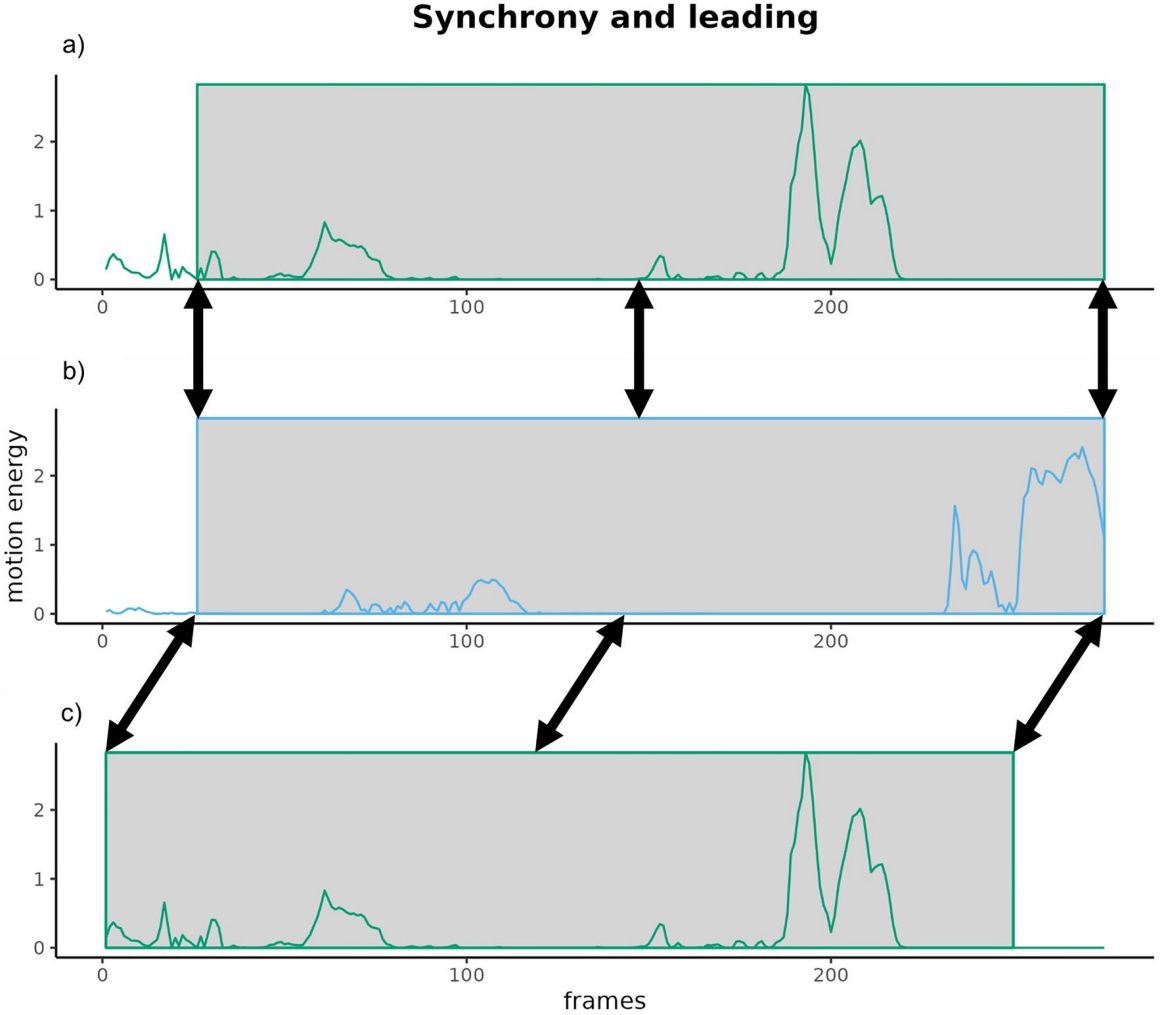
Interpersonal synchrony and leading based on motion quantity. This graph shows the motion energy, an estimate for motion quantity determined from video recordings, of two interactants, green and blue, and cross-correlation as a measurement for synchrony. Correlating (**a**) and (**b**) measures perfect alignment between the green and the blue interactaction partner with a lag of zero. By using a lag of 25 frames between (**b**) and (**c**), we can estimate how much motion energy of the green interaction partner influences motion energy of the blue interaction partner, i.e. how much green leads blue. Lags in the opposite direction would allow estimating the amount of leading that blue exerts on green. Windowed lagged cross-correlations measure synchrony in sliding windows by taking both perfect alignment and lagged behaviour into account, e.g., by averaging over all values or picking the peak value in the respective window.

Measures of interpersonal synchrony often make use of lagged cross-correlations between interactants’ behaviours^27,28^. Lagged cross-correlations not only provide measurements of perfect coordination but also when one person leads the other person^29^ (Fig. 1). Here, interpersonal synchrony is composed of perfect coordination and the leading of each interactant. Dyads whose interaction elicit a high level of interpersonal synchrony may lead to both interaction partners making better impressions to observers than interactants whose behaviours are not in sync. Additionally, one person leading their interaction partner’s behaviour could influence first impressions of themselves and their partner. Therefore, reduced interpersonal synchrony could be one cause driving less favourable impressions, given that previous studies have shown that it is altered in interactions with autistic individuals (for a review, see^30^).

Apart from symptoms regarding communication and social interaction^31^, ASC is often associated by patterns of behaviour that are more restricted, repetitive or inflexible compared to the behaviour of non-autistic people. Therefore, it is not surprising that research has shown movement atypicalities in autistic compared to non-autistic individuals^32^ and that people with ASC consider these to be common symptoms^33^. A review by Gowen and Hamilton^34^ suggests problems with integration of information for motor planning and an increased variance in motor behaviour. Additionally, a meta-analysis shows significant and substantial deficits in motor coordination in autistic compared to non-autistic individuals^35^. Importantly, Torres and colleagues^36^ argue that micro-movements could be a driving factor for differences which is consistent with a study showing less minimisation of jerking movements in people with ASC^37^. Interpersonal synchrony depends on micro-movements and is harder to achieve with an interaction partner who exhibits more jerking movements.

Interpersonal synchrony of motion, and its relevance for ASC, has been studied with various tasks ranging from synchronisation with objects to interpersonal synchrony of motion in natural interaction between individuals with and without ASC^30^. The extent of a person’s motion can be estimated by recording a video and analyzing the change of intensity values in any pixel. The more a person moves, the more does the intensity and color of the pixels change. Pixel value changes can be added up frame by frame resulting in a quantitative measure, the so called motion energy^28^. Georgescu and colleagues^38^ used motion energy and compared homogeneous autistic, homogeneous non-autistic and heterogeneous dyads consisting of one autistic and one non-autistic interactant. All dyads involving autistic participants were less synchronised compared to homogeneous non-autistic dyads, despite comparable overall motion in all three dyad compositions. Importantly, reduced interpersonal synchrony of motion energy seems to be specific for ASC in comparison to a clinical control group with differences in social interaction^39^. This robust effect led to the application of machine learning techniques to differentiate between autistic and non-autistic individuals based on interpersonal synchrony in motion energy^40^. Interpersonal synchrony of motion energy comprises situations of perfect coordination of movements but also of one person leading the other who adapts their motion energy to them. A recent study used this adaptation of motion energy and facial expressions to successfully train a support vector machine classifier to distinguish between autistic and non-autistic interactions with 79.5% balanced accuracy^40^; showing that synchrony of both modalities differs between homogeneous non-autistic dyads and heterogeneous dyads consisting of an autistic and non-autistic person.

### Aims and hypotheses of the present study

Given the close association of interpersonal synchrony and regular use of the rapport to assess impression formation, this study aimed to investigate the contribution of interpersonal synchrony of motion energy to the altered impression formation of people with ASC. Interpersonal synchrony, by definition, is a measurement that takes more than one person’s actions into account. However, it is possible to deconstruct interpersonal synchrony to focus on the individuals’ leading each other in an interaction. Given that previous research has established a link between synchrony and power perception^41^, we were interested in the influence of one interactant leading the other, as well as being led by their interaction partner, on impression formation. We created silent videos, which show solely the outlines of two people interacting, and asked non-autistic participants to rate their impression of one of the interactants. This approach allowed us to directly measure the mere effect of body motions in an interaction on impression ratings. Specifically, we were interested in the influence of each interactant’s motion energy on the other interactant, thereby assessing leading of each other’s motion energy. Based on prior impression formation and interpersonal synchrony research, we proposed the following hypotheses. First, we expected to replicate the effect of ASC on the ratings, such that interactants with ASC are rated less favourably than interactants without ASC (H1). Second, we hypothesised that interpersonal synchrony of motion energy between the two interactants, such that one person leads the other, would significantly influence the impression judgement of the leading person (H2). Third, we anticipated that diagnostic status might interact with this influence (H3). Furthermore, we observed participants’ eye movements to assess whether they focused on the target interactant whose impression they were asked to judge^42^. We considered that diagnostic status might influence gaze, such that autistic interactants lead to increased fixations due to idiosyncratic gestures associated with ASC (H4). In addition to our explicit hypotheses H1 to H4, we explored how autism-like traits in raters may influence the impression formation of interactants with and without ASC, as well as differences in nuanced aspects of impression formation, e.g., judging whether a person is likeable or trustworthy.

## Methods

This study was preregistered on OSF: https://osf.io/wtm3q. We added a hypothesis regarding the interaction effect of diagnostic status and synchrony on impression (H3) to the preregistered hypotheses. Concerning the analysis, we applied a Bayesian framework to allow for interpretation of the evidence *for* and *against* a hypothesis. Stimulus evaluation, preprocessing and analysis were performed with R *4.2.2*^43^ in RStudio *2022.12.0*^44^ and Python *3.9.13* in Spyder *5.3.3*^45^. Plots have been created with ggplot2 *3.4.2*^46^ and GIMP *2.10.34*^47^. All code used in the analysis of the stimuli, the pilot and the experimental data is available on OSF (https://osf.io/whgx6/).

### Participants

We aimed for a sample size of 195 non-autistic participants. The sample size was determined with a simulation-based power analysis as implemented in the R package mixedpower^48^ using pilot data from 37 participants. We determined a threshold of 2 and 1000 simulations to achieve 90% power for detecting an effect of interpersonal synchrony of motion energy on impression formation. Inclusion criteria were age between 18 and 60 years, no psychiatric or neurological diagnoses, normal or corrected-to-normal vision and informed consent. Exclusion criteria were high scores on the short form of German translation of the Autism Quotient questionnaire (above 5 of 10 points, AQ-10^49^) and low scores on a verbal intelligence test, the Wortschatz-Test (below 6 of 42 points, WST^50^). Additionally, we asked participants in a post-experimental debriefing questionnaire whether they have answered the questions conscientiously. They could either choose “Yes—my answers can be used for research without any problems” or “No—my answers should rather not be used”. This gave participants who were distracted or chose random ratings the opportunity to indicate that their data should not be used in the study. If they indicated that their data should not be used, their data was excluded from the analysis. We continuously preprocessed collected data along all inclusion and exclusion criteria. In total, we collected data from 247 participants of which one dataset was excluded because the participant advised against it and one because of a low WST score. Additionally, 49 participants scored above five in the AQ-10 (age: 18–59 years old, *mean* = 25.00 ± 4.59; 35 female). Therefore, 196 participants (age: 18–59 years old, *mean* = 26.56 ± 6.97; 142 female) were included to test H1, H2 and H3. For H4, we had to exclude additional participants due to insufficient gaze data quality, resulting in a sample of 91 participants (age: 19–59 years old, *mean* = 25.62 ± 5.93; 61 female). For more details on the sample, refer to the sample description in the supplementary materials. Participants were compensated for their participation with 10€ or course credit. This study was conducted following the Declaration of Helsinki and approved by the Ethics committee of the Medical Faculty, LMU Munich.

### Experimental procedure

**Figure 2:**
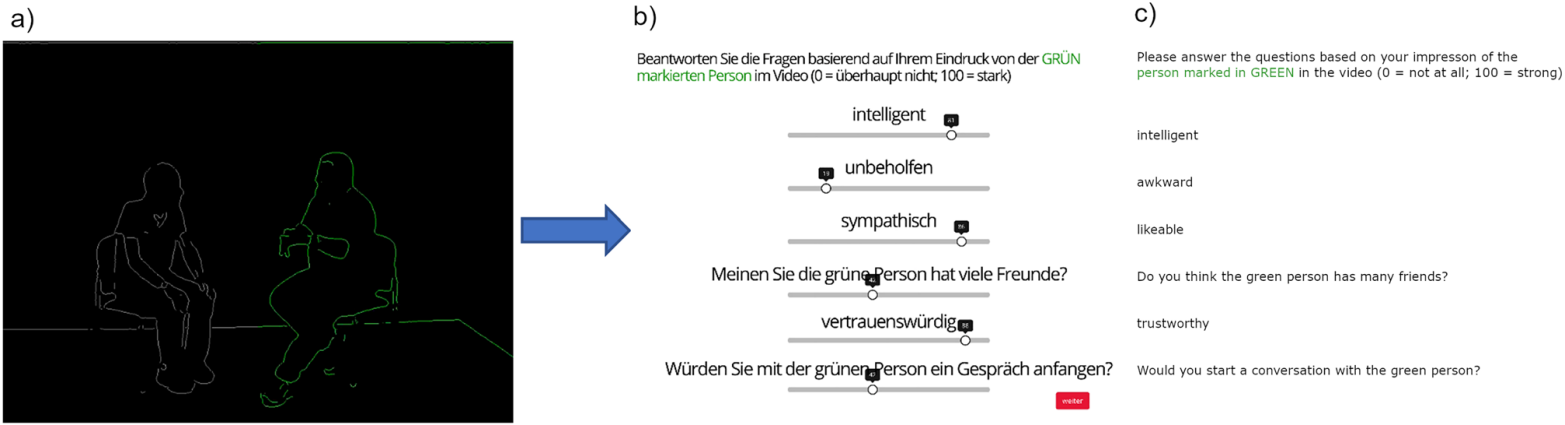
Task design. (**a**) Screenshot from one of the anonymised video stimuli shown to the participants with the target in green on the right side followed by (**b**) a screenshot of the rating scales and (**c**) the English translation.

Data collection was conducted in German using the Gorilla Experiment Builder on GORILLA™ (www.gorilla.sc), an online platform to create experiment designs^51^. The experiment consisted of study information, a consent page, demographic questions, AQ-10, WST, videos with their associated ratings (impression formation task) and a post-experiment debriefing questionnaire. In the impression formation task, participants saw 44 ten-second-long videos of two people interacting with each other. The videos did not contain any audio, and only anonymised outlines of the interactants were shown (Fig. 2). The outlines on half of the screen were coloured in green to indicate the target interactant, on whom participants should base their ratings. Each video was followed by six ratings on a scale from 0 (not at all) to 100 (very much): *intelligent*, *awkward*, *likeable*, *trustworthy*, *Would you start a conversation with the green person?* (abbr.: *conversation*) and *Do you think the green person has many friends?* (abbr.: *friends*; for the original German versions see Fig. 2). All but the last question were taken from Sasson et al.^5,8^. Participants had to respond to each rating to continue to the next trial. During the presentation of the videos, webcam-based eye tracking was used to measure gaze patterns. First, a calibration was performed. GORILLA™ uses support vector machines to track the participant’s face and, specifically, their eyes with a frequency of 60 Hz. For each 10-sec-long video, this should yield 600 samples under optimal conditions. Then, 2 m were calculated: the proportion of fixations on each of the screen halves and switches between the two halves.

### Stimulus creation

Stimuli were created from videos collected in previous studies investigating the influence of diagnostic status on interpersonal synchrony of motion energy in dyadic interactions. Specifically, 24 videos were excerpts from Georgescu et al.^38^, and 20 videos were excerpts from Koehler et al.^39^. Half of the videos showed two non-autistic interactants, in which one of the two interactants was highlighted green as the target to be judged. The other half of the videos presented mixed interactions between one autistic and one non-autistic interactant. In these videos, it was always the autistic interactant who was highlighted in green to be rated. Therefore, the design was balanced with regard to the independent variable diagnostic status (autistic, non-autistic).

Videos were muted and visually reduced to the outlines of the interactants in MATLAB *R2020b* (Natick, Massachusetts: The MathWorks Inc; Fig. 2). First, a Gaussian filter with *σ* = 2.0, as implemented in the imgaussfilt function, was applied to the greyscale versions of the videos in a frame-wise fashion to remove personally-identifying details that could be used to identify individuals. In two videos showing the same dyad, a Gaussian filter of *σ* = 2.5 was used which made the outlines slightly rougher but was hardly distinguishable from *σ* = 2.0 for the observer. Then, the edge function was used to determine edges in the filtered frames, so that only a rough outline of the individuals featured in the original video remained in the stimulus material. Last, one-half of the frames were coloured in green before exporting the edited video versions that finally were shown in the experiment. The green colouring indicated the target interactor who was to be rated.

Second, all videos were analysed using motion energy analyses (MEA)^28^. We focused on head motion because the videos from Koehler et al.^39^ included one person holding a clipboard, thereby artificially restricting motion in the upper body and arms. Resulting MEA values were further preprocessed in rMEA^52^. From each of the five-minute-long videos, two ten-second-long excerpts were chosen: one from 90 to 100 s, representing the introductory phase, and the other from 240 to 250 s, representing the body of the conversation. Outliers from the head region were removed using the rMEA::MEAoutlier function, which defines outliers as values more than ten times the standard deviation. Then, they were scaled using the rMEA::MEAscale function. Lastly, we computed lagged cross-correlations of the interactants’ MEA values within the same dyad using the rMEA::MEAccf function. We used pseudosynchrony to ensure that this measure captures interpersonal synchrony^53^. We evaluated (1) whether there is time dependency and (2) whether there exists synchrony in 10-s sections of the full videos as proposed by Moulder et al.^27^. Bayesian one-sample t-tests, as implemented by the BayesFactor::ttestBF function, revealed extreme evidence in favour of (1) and strong evidence in favor of (2), each based on 1000 iterations of pseudosynchrony per dyad (for details see the supplementary materials).

This investigation showed that lagged cross-correlation was an effective measure of interpersonal synchrony of motion energy in this sample. Additionally, lagged cross-correlations not only provide an estimate of the total interpersonal synchrony but also deconstructs the contributions of the two interactants by considering either lags where the target interactant is leading their interaction partner (*Green leading*) or vice versa (*White leading*). This approach has been successfully used to investigate relationship quality in the therapeutic context^29^. Since our participants were asked to rate only one interactant in each video, we decided to use these estimations of each interactant leading their counterpart as predictors in our analysis, rather than an interpersonal synchrony score describing the coordination of both interactants with each other. We log-transformed both leading scores to achieve normal distribution, which allows us to scale all our predictors.

Concerning the gaze patterns, we first excluded all trials where the face of the participant was tracked with 50% accuracy or less (value recommended by Gorilla Support, 2022^54^). Additionally, we set the minimum threshold for fixation duration to 50 ms. Trials with less than 400 samples were excluded from the analysis. One hundred and five participants with less than 50% of the trials left were excluded from the analysis of the gaze patterns resulting in a sample of 91 participants.

### Analysis

We used a combination of Bayesian linear mixed models, as implemented in the brms package^55^, and Bayesian t-tests, as implemented in the BayesFactor package^56^. For all random effects, we followed the guidelines by Barr et al.^57,58^. The Bayesian linear mixed models were run with four Markov chains with a total of 10,000 iterations each (50% warm-up). To draw conclusions on the significance of estimated parameters and differences, we used the brms::hypothesis function and Jeffrey’s scheme to interpret Bayes Factors^59^. The brms::hypothesis function computes an evidence ratio and a posterior probability under the hypothesis against its alternative. We chose one-sided testing in the direction of the respective estimate and adjusted *α* to 2.5%, since we preregistered non-directional testing with *α* = 0.05. Therefore, all parameter estimates with a posterior probability of above 97.5% for non-directional hypotheses (H1, H2, H3) and 95% for the directional hypothesis (H4) were considered significant.

For H1, H2 and H3, we averaged the six ratings to create a composite impression score to avoid multiple comparison correction by focusing on one outcome of interest for each hypothesis. Before computing the average, we reversed the the *awkwardness* rating, since here higher values correspond with a more negative impression. The unidim function of the psych package revealed a high factor fit of *fa.fit* = 0.98 suggesting that all ratings measure the same underlying concept. We entered the impression score into a Bayesian linear mixed model with three fixed effects of interest: diagnostic status (autistic, non-autistic), leading of the green target interactant (*Green leading*) and leading of the white non-target interactant (*White leading*). We also added three regressors of no interest: overall motion estimates of both interactants and the source of the videos^38,39^. All parametric predictors were scaled to allow for comparison of the estimates. Lastly, we added random intercepts for stimulus and participants, as well as random slopes for diagnosis and video source for the participants. We used treatment contrast coding^60^, with non-autistic interactants and videos from Georgescu et al.^38^ as the reference in relation to which all effects are evaluated.

Concerning the gaze patterns, we first used Bayesian paired t-tests to check whether participants focused on the green partner of the video led to increased fixation times. Then, we tested H4 by comparing fixation durations, as well as the number of switches per 100 samples, between the halves of the videos showing autistic and non-autistic targets for impression judgments. For each outcome variable, the interquartile method was used to detect and remove outliers.

For our explorative analyses, we performed similar Bayesian linear mixed models as described for testing H1, H2 and H3. We added binarised AQ-10 scores as a categorical predictor (0 = low, 1 = high) to the original model predicting the impression score to explore differences between participants with high and low autism-like traits. Additionally, we ran six Bayesian linear mixed models of the same structure for each of the six ratings to determine which nuanced aspects of impression formation are influenced by diagnostic status and the interactants leading one another.

## Results

### Influence of diagnostic status and leading on impression formation

**Figure 3:**
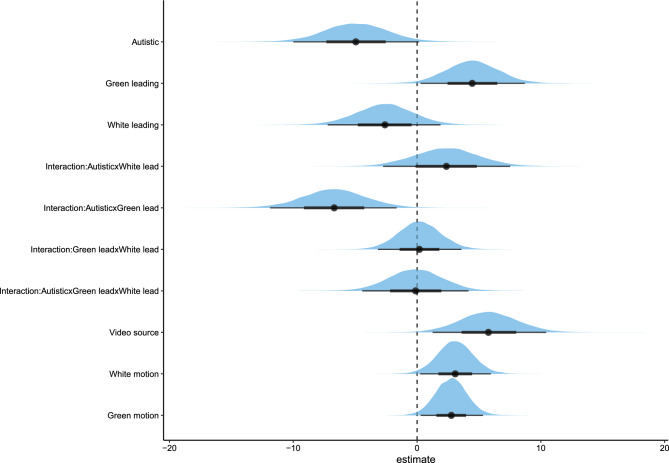
Distribution of the posterior samples. The distributions are shown for each predictor separately for the Bayesian linear mixed model testing H1, H2 and H3. Bolds and thin lines below the distributions show 95% and 66% of the distribution. The diagnosis ASC, the amount of the white person leading as well as the interaction between the amount of the green person leading with the diagnosis ASC had a negative effect on the impression score. Although, only the interaction effect was credible. The amount of the green person leading, the source of the video, motion of both white and green persons as well as the interaction between the amount of the white person leading and diagnosis ASC had a positive effect on impression score. However, evidence for the interaction was not credible. Both the interaction between green and white person leading and the three-way interaction had no clear effect on the impression score.

**Figure 4:**
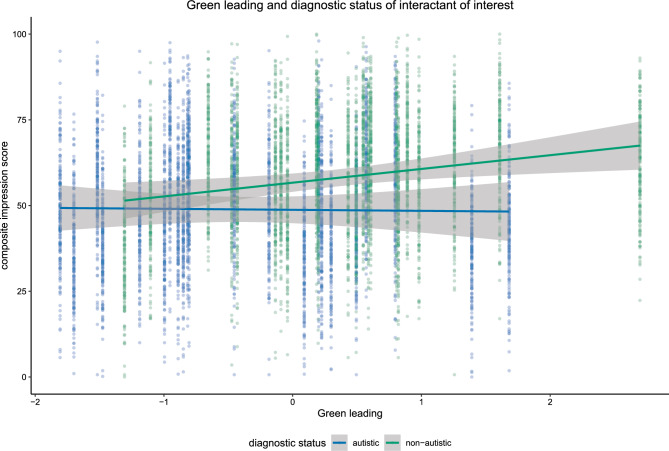
Relationship between leading and impression for autistic and non-autistic target interactants. Scatter plots show the relationship between the composite impression score and green leading in all trials. The regression line is based on the aggregated scores per video. While leading had a positive effect on impression scores of non-autistic interactants, there was no effect for autistic interactants.

The Bayesian linear mixed model estimated the influence of diagnostic status of the target interactant, leading of the target interactant (*Green leading*), leading of their interaction partner *(White leading*) and their interactions on the composite impression score of the rater (Fig. 3). Additionally, we added the influence of motion and video source to the model. Visual inspection suggests that autistic interactants were rated less favourably; however, evidence for H1 was not credible (*estimate* = − 4.93, *posterior probability* = 0.97). More leading by the target interactant led to better impression ratings; thus, confirming H2 (*Green leading*; *estimate* = 4.46, *posterior probability* = 0.98). This effect interacted with diagnostic status suggesting that ASC decreases the positive effect of leading on the impression score; thus, confirming H3 (*estimate* = − 6.70, *posterior probability* = 0.99; Fig. 4). For the full summary of the model, please see the supplementary materials). To further dissect the nature of the interaction, we divided the data into items featuring an autistic target interactant and items featuring a non-autistic target interactant. Then, we computed correlations between the composite impression scores and the amount of the target interactant separately for these two datasets. While there is a positive correlation between the composite impression scores and the amount of non-autistic target interactants leading (*r* = 0.55), there is no correlation between the composite impression scores and the amount of autistic target interactants leading (*r* = − 0.04). This reveals that leading only seems to improve impressions for non-autistic interactants.

### Gaze patterns during impression formation

First, we used a Bayesian paired samples t-test to assess whether participants focused on the green target interactant. There was extreme evidence that this was the case (*BF* = 1.19e+21), with participants fixating, on average, 64.95% of the time on the green half of the screen. However, neither the fixation proportion nor the switches between the two halves of the screen differed due to the diagnostic status. In fact, there was moderate evidence that diagnostic status does not affect the fixation proportion (*BF* = 0.29; *mean*_autistic_ = 65.00 ± 10.43%, *mean*_autistic_ = 64.00 ± 10.49%) or the number of switches per 100 samples (*BF* = 0.16; *mean*_autistic_ = 5.55 ± 1.73, *mean*_autistic_ = 5.49 ± 1.71%).

### Exploring the influence of autism-like traits on impression formation

We added another categorical predictor to the Bayesian linear mixed model distinguishing between participants with low and high autism-like traits. Participants with high autism-like traits were excluded from the linear mixed model testing H1, H2 and H3. Therefore, this exploratory analysis includes 245 participants, of which 49 were in the high AQ-10 group. While there were no differences between the two groups (*estimate* = 0.83, *posterior probability* = 0.75), there was a trend for the interaction effect of the AQ-10 group and diagnostic status (*estimate* = 1.81, *posterior probability* = 0.97), such that the people with high autism-like traits rated autistic and non-autistic interactants more similarly than people with low autism-like traits (Table 1).

**Table 1:** Mean and standard deviation for the impression score separated by the rater’s autism-like traits and diagnostic status of the observed target interactants.

	Autistic interactant	Non-autistic interactant	Difference
Low autism-like traits	48.86 ± 17.18	58.10 ± 16.47	9.24
High autism-like traits	51.18 ± 15.94	58.96 ± 15.05	7.78

### Exploring nuanced aspects of impression

We combined six ratings of different nuanced aspects of impression into one impression score to test our hypotheses. Additionally, we explored the effects of our predictors on the separate scores with six Bayesian linear mixed models (Table 2). While the ratings *awkward* and *friends* were crediblely predicted by diagnostic status with autistic interactants being rated less favourably, the other ratings depended on the green person leading and the diagnostic status in interaction with the green person leading. This means that the ratings *intelligent, likeable, conversation,* and *trustworthy* were higher when the target interactant was leading, but this effect was decreased or even fully diminished for autistic interactants. Additionally, the rating *trustworthy* depended on the white person leading, with higher values corresponding to the target interactant being judged as less trustworthy.

**Table 2:** Estimates for all explorative Bayesian linear mixed models with the nuanced aspects of the impression rating as outcomes. Asterisks mark credible estimates.

	Awkward	Friends	Intelligent	Likeable	Conversation	Trustworthy
Diagnostic status	− 12.51*	− 7.51*	− 1.96	− 2.33	-3.4	− 2.26
Green leading	2.07	2.46	6.03*	5.08*	5.79*	5.68*
White leading	0.82	− 0.56	− 2.71	− 4.34	− 4.07	− 4.92*
Diagnostic × green leading	− 8.14	− 5.92	− 7.94*	− 5.53*	− 7.2*	− 5.92*
Diagnostic × white leading	1.55	0.21	2.68	2.85	2.94	4.21
Diagnostic × green leading × white leading	3.13	− 1.44	0.96	− 1.54	− 0.91	− 0.58

## Discussion

When non-autistic individuals form first impressions of autistic individuals, they often judge them less favourably compared to non-autistic people^5,8,9^. Since autistic and non-autistic behaviour varies on multiple levels, it is paramount to carve out which behaviours might lead to less favourable impressions. In the current study, we collected first impressions of non-autistic raters based on ten-second-long videos. These videos showed a dyadic interaction in which only the outlines of the interactants were visible. Therefore, the only information participants could use to form their impressions of the target interactant was based on their motion. We analysed motion energy in these videos and computed lagged cross-correlations to measure how much each interactant led the other. This allowed us to assess the interactant’s individual contributions to interpersonal synchrony of motion energy. We found that overall impression was crediblely predicted by the extent to which the target interactant led their counterpart in the social interaction, i.e. how strongly their motion energy influenced the motion energy of their interaction partner. Yet, impression, as a result of leading, was only improved for non-autistic interactants, whereas leading did not influence the impression formation for autistic interactants. The previously reported robust effect of diagnostic status^5,8,9^ was only observable as a trend in the current study. This further corroborates that the specific aspects of behaviour isolated in the current study are at least partly responsible for the less favourable impression non-autistic individuals derive from observing autistic interactants.

ASC is consistently associated with decreased levels of interpersonal synchrony ^24,30,38,39^, and this decrease in interpersonal synchrony seems to be an important factor in impression formation. This study has identified a key feature that explains altered first impressions between individuals with and without ASC by showing an association between aspects of interpersonal synchrony and impression formation. Specifially, it shows the importance of how much one person’s motion energy is leading their interaction partner’s motion energy in a dyadic interaction. However, it is important to note that upon closer inspection, the attribution of leading seems to have an effect only benefiting non-autistic individuals. In fact, the presently reported statistical interaction between diagnostic status and leading of motion energy on impression scores suggests that behaviours improving first impressions for non-autistic individuals do not have the same positive effect for individuals with ASC. Interestingly, this reduced beneficial effect of interpersonal synchrony on impressions shows parallels to the finding that link interpersonal synchrony and cognitive empathy. Koehne and colleagues showed that perceived interpersonal synchrony increased cognitive empathy in non-autistic but not in autistic participants^22^. Given that interpersonal synchrony has also been linked with increased levels of rapport^15,16,18,19^, it should be investigated whether beneficial effects of interpersonal synchrony on rapport also exist when people without ASC interact with people with ASC.

It is unlikely that the results obtained in the current study are merely due to a bias against individuals with ASC, given that our participants were not aware of the diagnostic status. In fact, less than 5% of participants guessed that the study’s topic was related to ASC at post-experimental debriefing. By reducing available information about motion, we showed the main effect of diagnostic status on impression formation shrunk to a trend^5,8,9^, which means that the effect of diagnostic status could arguably be explained by distinctive features of motion in ASC. Withholding information like facial expression or speech features has not been done before. In contrast, many previous studies used videos rich with information, including audio of the person speaking^5,8,9^. The vast literature on differences in speech patterns between autistic and non-autistic adults^61–64^ together with the decreased effect found in this study suggests that including audio could amplify differences in impression ratings.

Based on our results, it is unclear which further aspects of the video properties explain the remaining variance in impressions. In future studies, experimenters may want to include ratings of the videos showing only the target interactant, and, therefore, only half of the interaction. This would allow them to assess impressions based on *motion alone* instead of *motion in interaction* as we did in this study. Additionally, other interactive aspects of motion may explain variance in the impression scores between people with and without ASC. For example, Fujiwara and Daibo^65^ recently proposed distinguishing behaviour matching, referring to the similarity in body postures, from interactional synchrony, referring to simultaneous movements and interaction rhythms. They showed that only behaviour matching enhanced empathic accuracy but not interactional synchrony. Therefore, future studies should aim to carve out how other aspects of motion may influence impression formation, along with interpersonal synchrony.

In addition to testing our preregistered hypothesis regarding the overall impression score, we also explored how diagnostic status, the leading role and their interaction influence nuanced aspects of impression formation by evaluating the separate ratings. The overall impression was based on judging the target interactant’s *awkwardness*, *intelligence*, *likeability* and *trustworthiness*, as well as how many friends the participants believed them to have (*friends*) and whether or not they would like to strike up a conversation with them (*conversation*). Both the rating of *awkwardness* and *friends* were only crediblely predicted by diagnostic status, with autistic target interactants being rated less favourably compared to non-autistic target interactants. The effect of diagnostic status on *awkwardness* was the strongest effect present in this study: target interactants with ASC were on average judged 12.5 of 100 points more awkward than target interactants without ASC. The ratings of *intelligence*, *likeability*, *conversation* and *trustworthiness* were not crediblely predicted by diagnostic status. These four ratings were predicted by a positive effect of the target interactant leading and the interaction between leading by the target interactant and the diagnostic status where interactants with ASC did not benefit from leading. This paints a similar picture to the overall impression score and is in contrast to previous studies that found influences of diagnostic status, especially on *likeability* and *conversation* ratings^5^. However, these studies did not show outlines of people in dyadic interactions, nor did they investigate the influence of interpersonal synchrony of motion energy on impression ratings. *Trustworthiness* was the only rating that was negatively predicted by how much the target interactant was led by their counterpart. There are different possibilities that could underly this effect. First, the counterpart may have judged the interactant to be less trustworthy as well and may have adjusted their behaviour due to this impression. Second, the leading of the counterpart may have influenced how *trustworthy* the counterpart was perceived which in turn could have influenced the impression of *trustworthiness* of the target interactant. Future studies should aim to disentangle these and other possible explanations. Sasson and colleagues also found in their research that *trustworthiness* was rated differently than other aspects of impression which they argued could be due to it being a character trait rather than social appeal or competence^5^. This explorative analysis shows that not all aspects of impression formation are influenced in the same way by diagnostic status and leading. Additionally, it shows a strong effect of aspects of interpersonal synchrony impacting impression formation, particularly leading by the target interactant.

Participants in our study were always asked to rate their impression of one of two people in a dyadic interaction. We captured their gaze with webcam-based eye tracking to ensure that they focus on the target interactant. Our data shows that participants spent nearly two-thirds of the time fixating on the respective target field. Therefore, we are confident that they focused more on the target interactant, while still taking into account the full interaction, when giving their impression judgements. In addition to the relationship between diagnostic status and impression formation, we expected that diagnostic status would influence gaze patterns. However, neither fixation duration, nor switches between the two interactants were crediblely predicted by diagnostic status. Based on this study, it is unclear whether this is due to the low spatial resolution of webcam-based eye-tracking or whether diagnostic status does not yield different gaze patterns. It is possible that more fine-grained gaze patterns would differ between forming an impression of an autistic or non-autistic individual and, thus, should be explored further.

Importantly, there are limitations to consider when interpreting the present results. First, webcam-based eye-tracking only offers rough estimates of gaze patterns because of coarse resolution and high data loss. In this study, we excluded more than half of the sample because gaze could not be reliably tracked for at least two thirds of the videos. Second, our sample was predominantly young (average 26.56 years old) and female (72.45%). Therefore, it is unclear if these effects extend to the general population. Future studies should aim for a more representative sample to ensure generalisability. Third, we used videos from two studies as source material for our stimuli^38,39^. Both studies assessed conversations of heterogeneous and homogeneous dyads, and their setups closely matched. However, they also differed: Georgescu et al.^38^ asked participants to engage in a five-minute, unstructured conversation planning a meal with foods and drinks that both participants dislike^66^. The participants were randomly paired adults with and without ASC who did not know each other before participating in the study. In contrast, Koehler et al.^39^ conducted diagnostic interviews with participants from a clinical population referred for autism diagnostics. In this case, participants were always paired with the first author of the paper who was holding a clipboard. Autistic interaction partners in the videos were those who received an ASC diagnosis, wherein an ASC diagnosis was ruled out for clinical control interaction partners^67^. To account for these differences, we added video source as a regressor to our models and found that interactants in videos from Koehler et al.^39^ were rated more favourably than interactants in videos from Georgescu et al.^38^. Fourth, the videos we used were reduced to outlines of two people engaging in conversations. Therefore, participants had limited information about the social interactions and the people engaged in them. This has the advantage that all effects found in this study can be traced back to the motion of two people interacting. However, this limits generalisability of these effects to more rich and natural social interactions. Last, non-autistic target interactants in our study were always part of an interaction of two non-autistic people while autistic people were always interacting with a non-autistic person. This means that not only the diagnostic status of the target interactants differed, but also their interaction partner. While fixations show that participants focused mainly on the target interactant, they also attended to their counterparts. Therefore, future studies should also include ratings of non-autistic target interactants who are part of a mixed interaction consisting of one autistic and one non-autistic partner as well as ratings of autistic target interactants who are part of a non-mixed interaction consisting of two autistic partners. This would allow further investigation of the influence of neurotype-matching which has been shown to influence rapport^68^ and interaction partners’ impression formation of each other^6^.

In this study, we investigated the link between interpersonal synchrony and impression formation of autistic and non-autistic people. We measured interpersonal synchrony by using lagged cross-correlations to estimate the amount of leading of each interactant in a dyadic conversation. While there was no credible overall difference between impressions formed of autistic and non-autistic target interactants, impressions were more favourable when the target interactant led their counterpart. This beneficial effect of leading only applied to non-autistic target interactants, suggesting that less favourable impressions of autistic people could be due to differences in interpersonal synchrony.

### Supplementary Information

Supplementary Information.

## Data Availability

Data and scripts to reproduce the results are available in the Open Science Framework repository: https://osf.io/whgx6/.
