# Supplementary material for: The role of interpersonal synchrony in forming impressions of autistic and non-autistic adults
Source: Sci Rep. 2023 Sep 18;13:15306. doi: 10.1038/s41598-023-42006-3 (PMC10507088; doi:10.1038/s41598-023-42006-3)
Supplement: Supplementary file 1 — Supplementary Information. [file 41598_2023_42006_MOESM1_ESM.pdf]

The role of interpersonal synchrony in forming impressions of autistic and non-autistic adults

Plank, I. S., Traiger, L. S., Nelson, A. M., Koehler, J. C., Lang, S. F.,
Tepest, R., Vogeley, K., Georgescu, A. L., Falter-Wagner, C. M.

24/06/2022

General information

This R Markdown document analyses pseudosynchrony of the videos used in the IMPAS study. It uses two approaches described by Moulder et al. (2018, *Psychol Methods*) to test the following hypotheses:

1. data shuffling: "There is no time dependency at all between these two time series."
2. section sliding: "Synchrony does not exist between sections of size *m* in these two time series"

However, we will not apply dyad shuffling, as the authors only recommended it for larger samples than the one we have here. Additionally, since the conversations had the same prompt, there may be similarities due to a similar structure. Dyad shuffling checks the following hypothesis: "The amount of synchrony between participants engaged in conversation with one another is no different than participants not in conversation with one another"

To run this script, one would need two .RData files, one containing the synchrony values used for the power estimation "stm_data.RData" and one with the complete synchrony values "stm_data_complete.RData". They are created by running "script_stim_ccf.R" and "script_stim_complete.R" respectively.

The following settings for the cross-correlation function were used (all in seconds): lag = 3 and both window size and increment = 10.

Data shuffling

Data shuffling is a kind of permutation test. From Moulder et al.: "For each pair of time series (X, Y) each data point, Xi, is randomly shuffled to create a new time series, Xs until no single data point existed at its original time point Xi!=Xjs.". This new time series is then paired with an unchanged time series Y.

In this script, this is done in twice for each dyad, once so that the left partner is unchanged and once so that the right partner is unchanged. For each, 500 permutations are computed. Note that no check for uniqueness is performed. In the end, an average for this specific video is calculated from the 2*500 pseudosynchrony values.

```r
dyad  = c()
psync = c()
ls.ds = list()

filename = "shuffle_d.RData"

if (!file.exists(filename)) {
  for (l in ls) {
    for (m in 1:length(l)) {
      d = strsplit(names(l)[m],"_")[[1]][2]
      dyad = c(dyad, d)
      val  = c()
      mea  = l[[m]]
      real = mea[["MEA"]]
      for (i in 1:n) {
        # shuffling left
        mea[["MEA"]][,1] = sample(mea[["MEA"]][,1])
        mea = MEAccf(mea, lagSec=lg, winSec=sz, incSec=sz)
        val = c(val,mea[["ccfRes"]][["grandAver"]])
        # shuffling right
        mea[["MEA"]][,1] = real[,1]
        mea[["MEA"]][,2] = sample(mea[["MEA"]][,2])
        mea = MEAccf(mea, lagSec=lg, winSec=sz, incSec=sz)
        val = c(val,mea[["ccfRes"]][["grandAver"]])
      }
      ls.ds = c(ls.ds,list(d,val))
      psync = c(psync, mean(val,na.rm=T))
    }
  }

  df.d_shuffle = data.frame(dyad,psync)
  save(df.d_shuffle,ls.ds,file=filename)
} else {
  load(filename)
}
```

Is there a time dependency between the time series?

```r
df.d_shuffle =
merge(df_sync[,c("dyad","all_lags_head","win_start")],df.d_shuffle,by="dyad",
all.x=T)

td = ttestBF(x = df.d_shuffle$all_lags_head,
        y = df.d_shuffle$psync,
        paired=T)

td

## Bayes factor analysis
## --------------
## [1] Alt., r=0.707 : 97739616 ±0%
```

```
##
## Against denominator:
##   Null, mu = 0
## ---
## Bayes factor type: BFoneSample, JZS
```

The paired t-test reveals extreme evidence in favour of a difference between the pseudosynchrony values obtained through data shuffling and the real synchrony values.

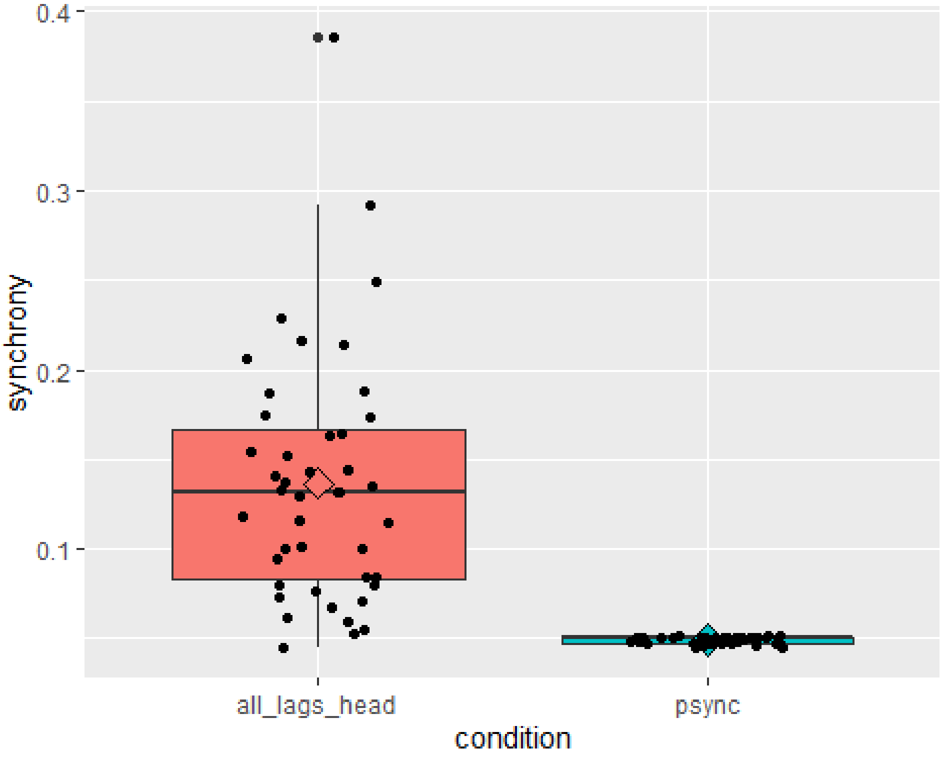

Figure 1: Difference between synchrony (all_lags_head) and pseudosynchrony (psync) obtained through data shuffling.

Segment shuffling

Segment shuffling, from Moulder et al., "requires researchers to cut a time series X into shorter sections of size m which are randomly appended to one another to create a new time series Xs until no section is in its original position". This new time series is then paired with an unchanged time series Y.

In this script, this is done in twice for each dyad, once so that the left partner is unchanged and once so that the right partner is unchanged. For each, 500 permutations with 10-second segments are computed. Note that no check for uniqueness is performed. In the end, an average for this specific video is calculated from the 2*500 pseudosynchrony values.

```
dyad  = c()
psync = c()
```

```r
ls.ss = list()

filename = "shuffle_s.RData"

if (!file.exists(filename)) {
  for (l in ls) {
    for (m in 1:length(l)) {
      d = strsplit(names(l)[m],"_")[[1]][2]
      dyad = c(dyad, d)
      val  = c()
      mea  = l[[m]]
      ch_L = split(mea[["MEA"]][,1], floor(seq_along(mea[["MEA"]][,1])/sz))
      ch_R = split(mea[["MEA"]][,2], floor(seq_along(mea[["MEA"]][,2])/sz))
      for (i in 1:n) {
        # shuffling left
        mea[["MEA"]][,2] = unlist(ch_R)
        mea[["MEA"]][,1] = unlist(sample(ch_L))
        mea = MEAccf(mea, lagSec=lg, winSec=sz, incSec=sz)
        val = c(val,mea[["ccfRes"]][["grandAver"]])
        # shuffling right
        mea[["MEA"]][,1] = unlist(ch_L)
        mea[["MEA"]][,2] = unlist(sample(ch_R))
        mea = MEAccf(mea, lagSec=lg, winSec=sz, incSec=sz)
        val = c(val,mea[["ccfRes"]][["grandAver"]])
      }
      ls.ss = c(ls.ss,list(d,val))
      psync = c(psync, mean(val,na.rm=T))
    }
  }

  df.s_shuffle = data.frame(dyad,psync)
  save(df.s_shuffle,ls.ss,file=filename)
} else {
  load(filename)
}
```

Is there synchrony between sections of size 10 seconds in these two time series?

```r
df.s_shuffle =
merge(df_sync[,c("dyad","all_lags_head","win_start")],df.s_shuffle,by="dyad",
all.x=T)

ts = ttestBF(x = df.s_shuffle$all_lags_head,
        y = df.s_shuffle$psync,
        paired=T)

ts

## Bayes factor analysis
## --------------
## [1] Alt., r=0.707 : 10.83313 ±0%
```

```
##
## Against denominator:
##   Null, mu = 0
## ---
## Bayes factor type: BFoneSample, JZS
```

The paired t-test reveals strong evidence in favour of a difference between the pseudosynchrony values obtained through segment shuffling and the real synchrony values.

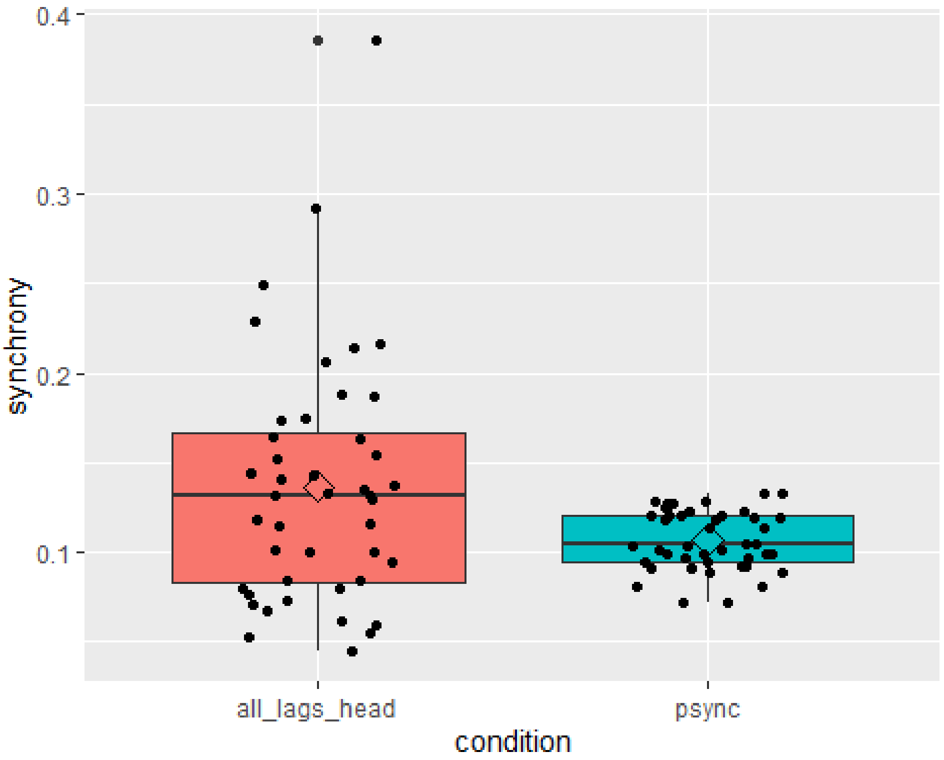

Figure 2: Difference between synchrony (all_lags_head) and pseudosynchrony (psync) obtained through segment shuffling.

Analysis

Software versions

```
R.Version()$version.string

## [1] "R version 4.2.2 (2022-10-31 ucrt)"

packageVersion('brms')

## [1] '2.18.0'

packageVersion('BayesFactor')
```

```
## [1] '0.9.12.4.4'

packageVersion('bayestestR')

## [1] '0.13.0'
```

Hypotheses

This study was preregistered after piloting 37 participants and before starting data collection. The goal was to analyse 195 datasets of people who have an AQ score below 6 and a WST score above 5.

We formulated the following hypotheses:

3. Autistic interactants are judged differently compared to non-autistic interactants (non-directional, preregistered).
4. Synchrony scores are associated with ratings (non-directional, preregistered).
5. The effect of synchrony interacts with the diagnostic status of the interactant (non-directional, *not* preregistered)
6. People fixate more often on autistic interactants due to idiosyncratic gestures associated with autism (directional, preregistered).

Additionally, we will investigate the interaction of leading and diagnostic status on ratings. We will use directional hypothesis testing in the direction of the estimate with an alpha of 2.5%, therefore, equivalent to a non-directional test with alpha set to 5% as preregistered.

Initialisation

First, let's load all the data that we are going to need to investigate these hypotheses.

```
# load the relevant data in long format
df = read_csv("data/df_all.csv", show_col_types = F) %>%
  mutate(sub_id = as.factor(sub_id)) %>%
  mutate_if(is.character, as.factor) %>%
  filter(WST >= 6) %>% # exclude people with IQ score that's too low
  # exclude people that say themselves the data should not be used
  filter(is.na(Seriousness_check) | Seriousness_check != "nein -  meine
Antworten sollten lieber nicht verwendet werden") %>%
  mutate(aq_high = as.factor(AQ > 5), # add group: high or low aq
         awk     = 100 - awk) %>% # awkwardness rating needs to be reversed
  rowwise() %>%
  mutate(
    imp = mean(c(int,lik,tru,awk,tlk,fri)),
    diagnosis = recode_factor(as.factor(diagnosis),
       "ASD" = "autistic",
       "TD" = "non-autistic"
    )
    ) %>%
  rename("diagnostic status" = "diagnosis")
# have a closer look at the video specific values
```

```r
df.sync = df %>% select(video, `diagnostic status`,
sync,green_lead,white_lead,
        mot_head,green_mot_head,white_mot_head) %>% distinct()
# first, let's check out our normal distributions of the predictors
# synchrony values are not normally distributed - but after log transforming
they are
df.sync = df.sync %>%
  mutate_at(vars(matches('sync|white_lead|green_lead')), log)
idx = grep("sync", colnames(df.sync))
lapply(df.sync[,idx:ncol(df.sync)], shapiro.test)
```

```
## $sync
##
##  Shapiro-Wilk normality test
##
## data:  X[[i]]
## W = 0.98897, p-value = 0.9453
##
##
## $green_lead
##
##  Shapiro-Wilk normality test
##
## data:  X[[i]]
## W = 0.98024, p-value = 0.6433
##
##
## $white_lead
##
##  Shapiro-Wilk normality test
##
## data:  X[[i]]
## W = 0.98358, p-value = 0.7761
##
##
## $mot_head
##
##  Shapiro-Wilk normality test
##
## data:  X[[i]]
## W = 0.96666, p-value = 0.2296
##
##
## $green_mot_head
##
##  Shapiro-Wilk normality test
##
## data:  X[[i]]
## W = 0.95324, p-value = 0.07266
##
```

```
##
## $white_mot_head
##
##  Shapiro-Wilk normality test
##
## data:  X[[i]]
## W = 0.95902, p-value = 0.1197

# now we can scale our predictors - Bodo Winter: "If in doubt, center"
df.sync[,idx:ncol(df.sync)] = scale(df.sync[,idx:ncol(df.sync)],scale=T)
# get rid of old values before merging data frames for analysis
df = df %>% select(-c(sync, green_lead, white_lead, mot_head, green_mot_head,
white_mot_head))
df = merge(df,df.sync)
write_csv(df.sync, file = "data/df_sync-used.csv")
rm(list = c("df.sync"))
# setting contrasts (sum coding)
df$place                    = as.factor(df$place)
df$`diagnostic status`  = as.factor(df$`diagnostic status`)
df$aq_high                  = as.factor(df$aq_high)
contrasts(df$aq_high)   = contr.treatment(2, base = 2)
contrasts(df$`diagnostic status`)   = contr.treatment(2, base = 2)
# turning sub and stm into simple numbers
df$sub = as.numeric(as.factor(df$sub_id))
df$stm = as.numeric(as.factor(df$video))

write_csv(df, "data/df_all_analysis.csv")
```

Sample description

```
df.sub = df %>% select(
  sub_id, aq_high, AQ, WST, Alter, Bildung, Deutschsprachig, Erkrankung,
Geschlecht,
  Muttersprache, Sehhilfe, Kontakt_ASD) %>%
  distinct()

kable(summary(df.sub[df.sub$aq_high == F,c(2:12)]))
```

aq_high	AQ	WST	Alter	Bildung	Deutschsprachig	Erkrankung	Geschlecht	Muttersprache	Sehhilfe	Kontakt_ASD
FALSE:196	Min. :0.000	Min. :15.00	Min. :18.00	Abitur/Fachabitur :78	1-3 Jahre : 4	nein: 196	männlich: 54	ja :184	Brille : 48	Min. : 0.00
TRUE : 0	1st Qu.: 1.000	1st Qu.: 31.00	1st Qu.: 23.00	Bachelor :48	10+ Jahre :183	NA	weiblich:142	nein: 12	keine benötigt:123	1st Qu.: 13.25
NA	Med	Med	Med	Diplom/	3-5	NA	NA	NA	Konta	Media

aq_high	AQ	WST	Alter	Bildung	Deutschsprachig	Erkrankung	Geschlecht	Muttersprache	Sehhilfe	Kontakt_ASD
	ian :2.000	ian :33.00	ian :24.00	Magister/ Master/ Staatsexamen :47	Jahre : 4				ktlinsen : 25	n : 33.00
NA	Mean :2.388	Mean :32.33	Mean :26.56	Abschluss einer anerkannten Berufsausbildung :11	5-10 Jahre : 2	NA	NA	NA	NA	Mean : 36.77
NA	3rd Qu.: 3.000	3rd Qu.: 35.00	3rd Qu.: 28.00	Meister-/Techniker- oder gleichwertiger Fachabschluss: 3	mehr als ein Jahr: 1	NA	NA	NA	NA	3rd Qu.: 58.00
NA	Max. :5.000	Max. :40.00	Max. :59.00	Promotion : 3	Niemals : 2	NA	NA	NA	NA	Max. :100.00
NA	NA	NA	NA	(Other) : 6	NA	NA	NA	NA	NA	NA's :18

```
kable(summary(df.sub[df.sub$aq_high == T,c(2:12)]))
```

Table 1: Demographic information describing the participants.

aq_high	AQ	WST	Alter	Bildung	Deutschsprachig	Erkrankung	Geschlecht	Muttersprache	Sehhilfe	Kontakt_ASD
FALSE : 0	Min. : 6.000	Min. :16.00	Min. :19.00	Abitur/ Fachabitur :21	1-3 Jahre : 3	nein: 49	männlich:14	ja :43	Brille :12	Min. : 0.00
TRUE	1st Qu.	1st Qu.:	1st Qu.:	Diplom/ Magister/	10+ Jahre	NA	weiblich:3	nein: 6	keine benöti	1st Qu.:1

aq_high	AQ	WST	Alter	Bildung	Deutschsprachig	Erkrankung	Geschlecht	Muttersprache	Sehhilfe	Kontakt_ASD
:49	: 7.000	32.00	23.00	Master/ Staatsexamen :15	:45		5		gt:30	1.00
NA	Median : 8.000	Median :34.00	Median :25.00	Bachelor :12	3-5 Jahre : 1	NA	NA	NA	Kontaktlinsen : 7	Median :32.50
NA	Mean : 7.633	Mean :32.69	Mean :25.63	Abschluss einer anerkannten Berufsausbildung : 1	5-10 Jahre : 0	NA	NA	NA	NA	Mean :37.61
NA	3rd Qu. : 9.000	3rd Qu.: 35.00	3rd Qu.: 27.00	Haupt-/Volksschulabschluss : 0	mehr als ein Jahr: 0	NA	NA	NA	NA	3rd Qu.:66.25
NA	Max. :10.000	Max. :38.00	Max. :44.00	Meister-/Techniker- oder gleichwertiger Fachabschluss: 0	Niemals : 0	NA	NA	NA	NA	Max. :91.00
NA	NA	NA	NA	(Other) : 0	NA	NA	NA	NA	NA	NA's :5

We asked our participants to guess our research question. Let's check how many guessed something with ASD:

```
df.rq = df %>% select(sub_id, `Was_untersucht?`) %>%
  distinct()

mean(grepl("autis|Autis",df.rq$`Was_untersucht?`))

## [1] 0.04489796
```

H1, H2, H3: diagnostic status of the interactant and synchrony values

The first two hypotheses and the (not preregistered) interaction can all be tested with one model. According to our hypotheses, we are interested in the influence of *diagnostic status* and *synchrony* which are both within-subjects. Since the participants are supposed to rate one of the two interactants, we will break down synchrony into two scores: *green_lead* and *white_lead*. These factors are computed with lagged cross-correlations to estimate the influence of one interactant's motion energy on the other interactant's motion energy. We include both *green_lead* and *white_lead* as well as all interactions as fixed effects. Additionally, we include three factors for which variance we want to control, but we are not interested in their influence per se: *green_mot_head*, *white_mot_head* and *place* which are also within-subjects.

Choosing the model structure

Stimuli and subjects are our random effects: "A random factor is a factor whose levels are considered to represent a proper subset of all the levels in the population. Usually, you treat a factor as random when the levels you have in the data are the result of sampling, and you want to generalize beyond those levels. […] Rather than estimating the intercept and slope for each [level of the random effect] without considering the estimates for [the other levels], the model estimates values for the population, and pulls the estimates for individual [levels] toward those values, a statistical phenomenon known as shrinkage. […] The tradition in psychology when performing confirmatory analyses is to use the maximal random effects structure justified by the design of your study". (Barr et al., 2021) Following Barr et al. (2021), we use the following guidelines to determine the nature of the random effects:

(1) If there are repeated measures on sampling units, you need a random intercept for that random factor: (1 | unit_id);
(2) If a factor x is between-unit, you do not need a random slope for that factor;
(3) Determine the highest order interaction of within-subject factors for the unit under consideration. If you have pseudoreplications within each cell defined by those combinations (i.e., multiple observations per cell), then for that unit you will need a slope for that interaction as well as for all lower order effects. If there are no pseudoreplications, then you do not need any random slopes.

According to (1), we need to add random intercepts to both stimuli and subjects: there are multiple stimuli per subject and multiple subjects per stimuli. The factors *diagnostic status*, *green_lead*, *white_lead*, *green_mot_head*, *white_mot_head* and *place* are all between-**stimuli** (one stimulus has one level), therefore, we don't need any random slope for the random effect stimuli according to (2). However, they are all within-**subjects** (one subjects gets every level), so we need to have a look at their interactions and what is the highest order interaction that has pseudoreplications. The highest order interaction of these factors has only one measurement per subject associated with it, so there are no pseudoreplications. This is because each stimulus is only seen once per subject and each stimulus is associated with a unique *green_lead*, *white_lead*, *green_mot_head* and *white_mot_head* value. However,

there are pseudoreplications of *place* and *diagnostic status*, so we add random slopes for these factors.

Unidimensionality analysis of the ratings

Instead of testing all six ratings separately, we will first investigate whether they might all measure the same concept, a composite impression score.

```
unidim(df[,c("awk","lik","tru","int","tlk","fri")])

##
## A measure of unidimensionality
##  Call: unidim(x = df[, c("awk", "lik", "tru", "int", "tlk", "fri")])
##
## Unidimensionality index =
##        u av.r fit   fa.fit    alpha     av.r median.r Unidim.A
##     0.87     0.88     0.98     0.86     0.50     0.56     1.00
##
## unidim adjusted index reverses negatively scored items.
## alpha    Based upon reverse scoring some items.
## average and median  correlations are based upon reversed scored items
```

Reasonably high factor fit of 0.98 suggests that the variables might measure the same concept. Therefore, we will test our hypotheses with this composite score.

Investigate distribution of the outcome

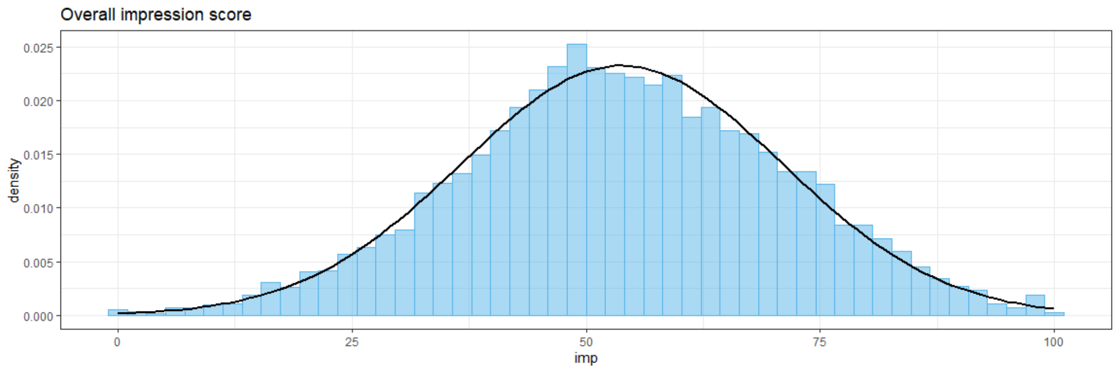

Figure 3: Distribution of the composite score integrating all ratings to an impression score.

Low AQ group: composite score

Model summary and assumptions check

```
# marginal R2 describes the proportion of variance explained by the fixed
factor(s)
# conditional R2 describes the proportion of variance explained by both the
fixed and random factors
if (file.exists("models/brm_imp.rds")) {
  mimp = readRDS('models/brm_imp.rds')
} else {
```

```
  mimp = brm(formula = imp ~
               `diagnostic status`* green_lead * white_lead + # fixed effects
               green_mot_head + white_mot_head + place + # regressors of no
interest
               (`diagnostic status`+ place|sub) + (1|stm), # random effects
             data = df[df$aq_high == F,],
             control = list(adapt_delta = 0.99,
                            max_treedepth = 13),
             save_pars = save_pars(all = T),
             chains = 4,
             warmup = nw, iter = nw*2)
  saveRDS(mimp, file = 'models/brm_imp.rds')
}

summary(mimp)

##  Family: gaussian
##   Links: mu = identity; sigma = identity
## Formula: imp ~ diagnosis * green_lead * white_lead + green_mot_head +
white_mot_head + place + (diagnosis + place | sub) + (1 | stm)
##    Data: df[df$aq_high == F, ] (Number of observations: 8624)
##   Draws: 4 chains, each with iter = 10000; warmup = 5000; thin = 1;
##          total post-warmup draws = 20000
##
## Group-Level Effects:
## ~stm (Number of levels: 44)
##               Estimate Est.Error l-95% CI u-95% CI Rhat Bulk_ESS Tail_ESS
## sd(Intercept)     6.32      0.83     4.93     8.15 1.00     6755    11241
##
## ~sub (Number of levels: 196)
##                             Estimate Est.Error l-95% CI u-95% CI Rhat
Bulk_ESS
## sd(Intercept)                   7.71      0.46     6.85     8.65 1.00
6850
## sd(diagnosis1)                  4.37      0.41     3.59     5.21 1.00
7434
## sd(placeMN)                     4.04      0.40     3.27     4.84 1.00
8441
## cor(Intercept,diagnosis1)      -0.17      0.10    -0.36     0.03 1.00
11205
## cor(Intercept,placeMN)         -0.10      0.10    -0.30     0.11 1.00
11825
## cor(diagnosis1,placeMN)        -0.28      0.13    -0.52    -0.02 1.00
5661
##                             Tail_ESS
## sd(Intercept)                  10249
## sd(diagnosis1)                 12807
## sd(placeMN)                    13543
## cor(Intercept,diagnosis1)      13745
## cor(Intercept,placeMN)         14471
```

```
## cor(diagnosis1,placeMN)       9044
##
## Population-Level Effects:
##                                Estimate Est.Error l-95% CI u-95% CI Rhat
## Intercept                         52.32      2.29    47.76    56.82 1.00
## diagnosis1                        -4.93      2.57   -10.00     0.15 1.00
## green_lead                         4.46      2.13     0.29     8.70 1.00
## white_lead                        -2.61      2.30    -7.22     1.90 1.00
## green_mot_head                     2.77      1.28     0.29     5.32 1.00
## white_mot_head                     3.09      1.44     0.25     5.96 1.00
## placeMN                            5.80      2.35     1.26    10.43 1.00
## diagnosis1:green_lead             -6.70      2.58   -11.87    -1.65 1.00
## diagnosis1:white_lead              2.36      2.61    -2.74     7.52 1.00
## green_lead:white_lead              0.20      1.71    -3.15     3.58 1.00
## diagnosis1:green_lead:white_lead  -0.12      2.19    -4.42     4.15 1.00
##                                Bulk_ESS Tail_ESS
## Intercept                          5580     8212
## diagnosis1                         6206    10054
## green_lead                         6398     9343
## white_lead                         6230     9124
## green_mot_head                     6653     9297
## white_mot_head                     6157     9133
## placeMN                            6533     9622
## diagnosis1:green_lead              6963     9505
## diagnosis1:white_lead              6771    10137
## green_lead:white_lead              7322    10777
## diagnosis1:green_lead:white_lead   7779    11230
##
## Family Specific Parameters:
##       Estimate Est.Error l-95% CI u-95% CI Rhat Bulk_ESS Tail_ESS
## sigma    13.01      0.10    12.81    13.21 1.00    32015    14279
##
## Draws were sampled using sampling(NUTS). For each parameter, Bulk_ESS
## and Tail_ESS are effective sample size measures, and Rhat is the potential
## scale reduction factor on split chains (at convergence, Rhat = 1).

# check assumptions for this model
check_model(mimp,
check=c("qq","linearity","homogeneity","reqq","vif","pp_check"))
```

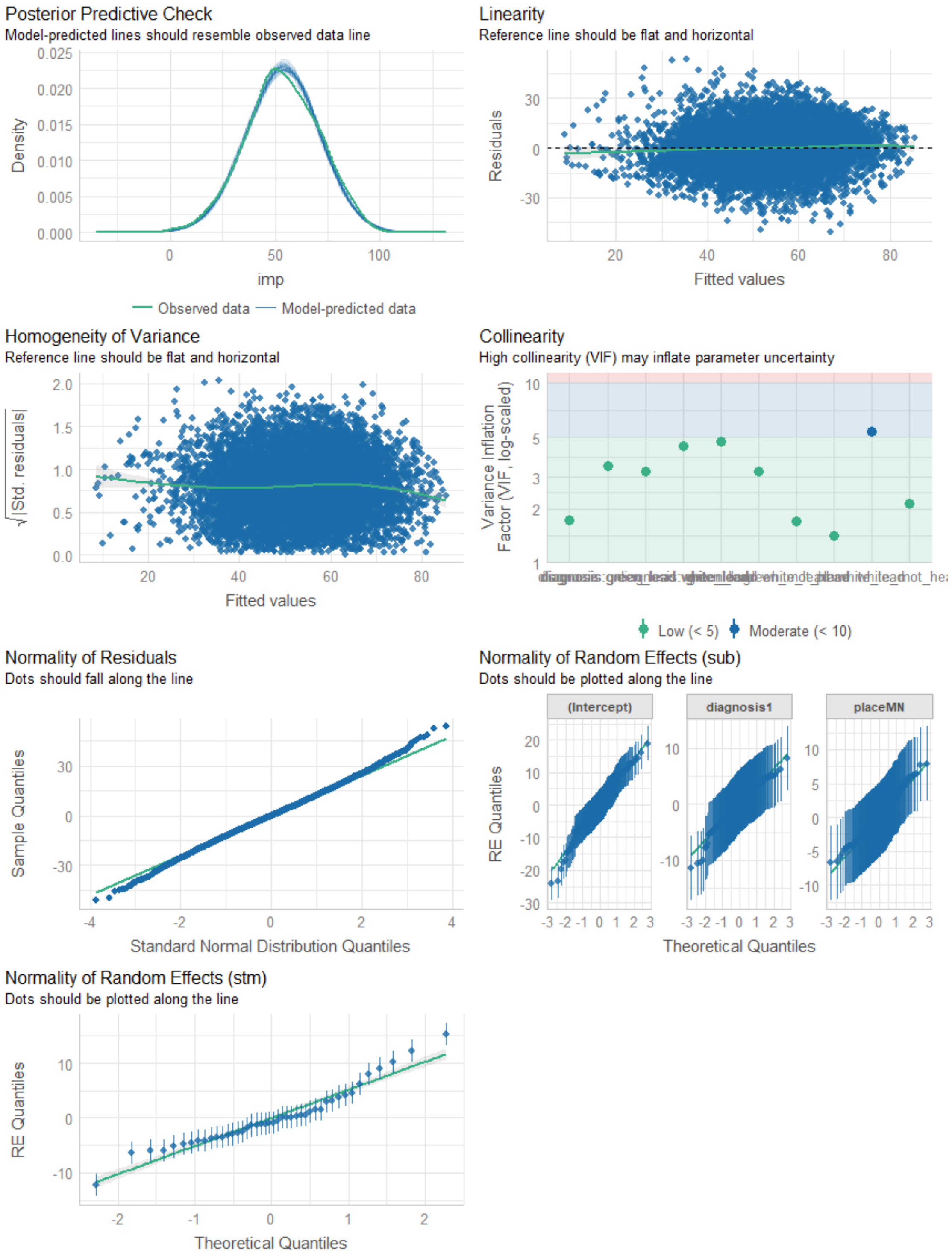

Figure 4: Checking the assumptions of the Bayesian linear mixed model investigating the predictors of the composite impression score.

Plot estimates and test hypotheses

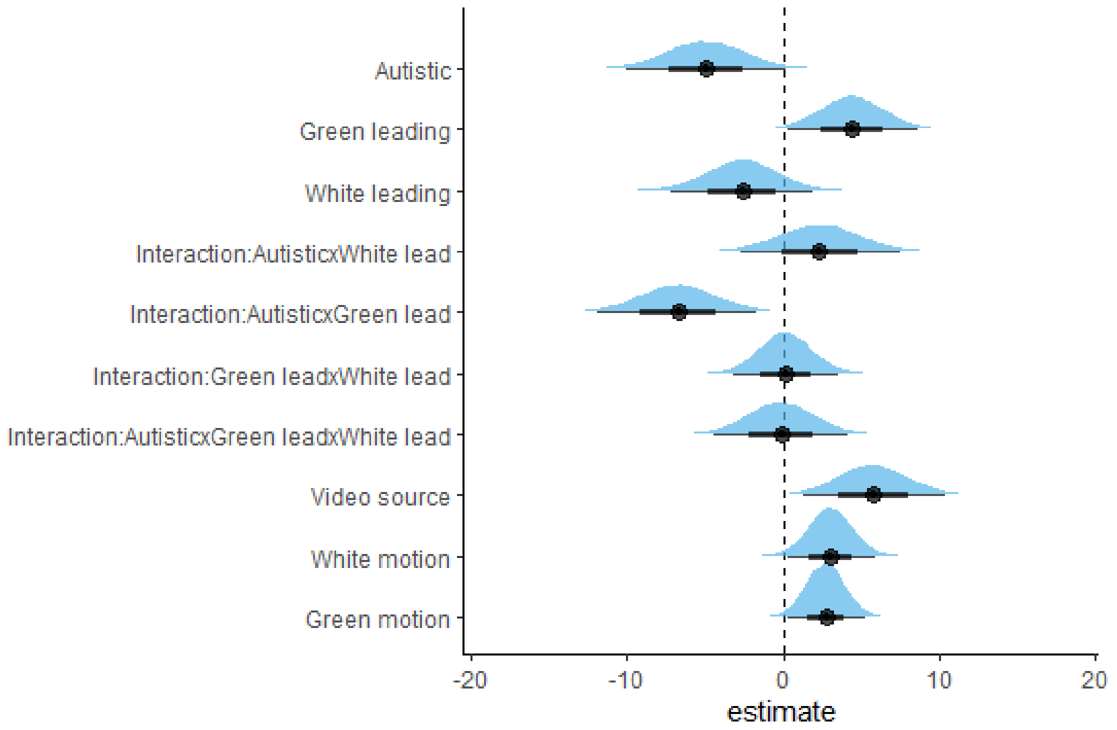

Figure 5: Plot the estimates of the predictors of the composite impression score.

```
# H1: effect of autism diagnosis
hypothesis(mimp, "diagnosis1 < 0", alpha = 0.025)

## Hypothesis Tests for class b:
##          Hypothesis Estimate Est.Error CI.Lower CI.Upper Evid.Ratio
Post.Prob
## 1 (diagnosis1) < 0    -4.93      2.57      -10     0.15      34.27
0.97
##   Star
## 1
## ---
## 'CI': 95%-CI for one-sided and 97.5%-CI for two-sided hypotheses.
## '*': For one-sided hypotheses, the posterior probability exceeds 97.5%;
## for two-sided hypotheses, the value tested against lies outside the 97.5%-
CI.
## Posterior probabilities of point hypotheses assume equal prior
probabilities.

# H2: effect of synchrony
hypothesis(mimp, "green_lead > 0", alpha = 0.025) # for non-autistic

## Hypothesis Tests for class b:
##          Hypothesis Estimate Est.Error CI.Lower CI.Upper Evid.Ratio
Post.Prob
## 1 (green_lead) > 0     4.46      2.13     0.29      8.7      53.79
```

```
0.98
##   Star
## 1    *
## ---
## 'CI': 95%-CI for one-sided and 97.5%-CI for two-sided hypotheses.
## '*': For one-sided hypotheses, the posterior probability exceeds 97.5%;
## for two-sided hypotheses, the value tested against lies outside the 97.5%-
CI.
## Posterior probabilities of point hypotheses assume equal prior
probabilities.
```

```
hypothesis(mimp, "white_lead < 0", alpha = 0.025) # for non-autistic
```

```
## Hypothesis Tests for class b:
##         Hypothesis Estimate Est.Error CI.Lower CI.Upper Evid.Ratio
Post.Prob
## 1 (white_lead) < 0    -2.61       2.3    -7.22      1.9       7.08
0.88
##   Star
## 1
## ---
## 'CI': 95%-CI for one-sided and 97.5%-CI for two-sided hypotheses.
## '*': For one-sided hypotheses, the posterior probability exceeds 97.5%;
## for two-sided hypotheses, the value tested against lies outside the 97.5%-
CI.
## Posterior probabilities of point hypotheses assume equal prior
probabilities.
```

```
# H3
hypothesis(mimp, "diagnosis1:green_lead < 0", alpha = 0.025)
```

```
## Hypothesis Tests for class b:
##                 Hypothesis Estimate Est.Error CI.Lower CI.Upper Evid.Ratio
## 1 (diagnosis1:green... < 0     -6.7      2.58   -11.87    -1.65     185.92
##   Post.Prob Star
## 1      0.99    *
## ---
## 'CI': 95%-CI for one-sided and 97.5%-CI for two-sided hypotheses.
## '*': For one-sided hypotheses, the posterior probability exceeds 97.5%;
## for two-sided hypotheses, the value tested against lies outside the 97.5%-
CI.
## Posterior probabilities of point hypotheses assume equal prior
probabilities.
```

```
hypothesis(mimp, "diagnosis1:white_lead > 0", alpha = 0.025)
```

```
## Hypothesis Tests for class b:
##                 Hypothesis Estimate Est.Error CI.Lower CI.Upper Evid.Ratio
## 1 (diagnosis1:white... > 0     2.36      2.61    -2.74     7.52        4.5
##   Post.Prob Star
## 1      0.82
```

```
## ---
## 'CI': 95%-CI for one-sided and 97.5%-CI for two-sided hypotheses.
## '*': For one-sided hypotheses, the posterior probability exceeds 97.5%;
## for two-sided hypotheses, the value tested against lies outside the 97.5%-
CI.
## Posterior probabilities of point hypotheses assume equal prior
probabilities.
```

```
hypothesis(mimp, "diagnosis1:green_lead:white_lead < 0", alpha = 0.025)
```

```
## Hypothesis Tests for class b:
##                 Hypothesis Estimate Est.Error CI.Lower CI.Upper Evid.Ratio
## 1 (diagnosis1:green... < 0    -0.12      2.19    -4.42     4.15        1.1
##   Post.Prob Star
## 1      0.52
## ---
## 'CI': 95%-CI for one-sided and 97.5%-CI for two-sided hypotheses.
## '*': For one-sided hypotheses, the posterior probability exceeds 97.5%;
## for two-sided hypotheses, the value tested against lies outside the 97.5%-
CI.
## Posterior probabilities of point hypotheses assume equal prior
probabilities.
```

As we can see, there is an interaction between *diagnostic status* and *green_lead*. We will compute correlations to investigate this interaction further. We also plot the data with a linear model line added to it.

```
## `summarise()` has grouped output by 'video'. You can override using the
## `.groups` argument.

##
##  Pearson's product-moment correlation
##
## data:  df.agg[df.agg$`diagnostic status` == "autistic", ]$imp and
df.agg[df.agg$`diagnostic status` == "autistic", ]$green_lead
## t = -0.16349, df = 20, p-value = 0.8718
## alternative hypothesis: true correlation is not equal to 0
## 95 percent confidence interval:
##  -0.4511928  0.3910985
## sample estimates:
##         cor
## -0.03653411

##
##  Pearson's product-moment correlation
##
## data:  df.agg[df.agg$`diagnostic status` == "non-autistic", ]$imp and
df.agg[df.agg$`diagnostic status` == "non-autistic", ]$green_lead
## t = 2.9775, df = 20, p-value = 0.007442
## alternative hypothesis: true correlation is not equal to 0
## 95 percent confidence interval:
```

```
##  0.1730182 0.7909895
## sample estimates:
##    cor
## 0.5542
```

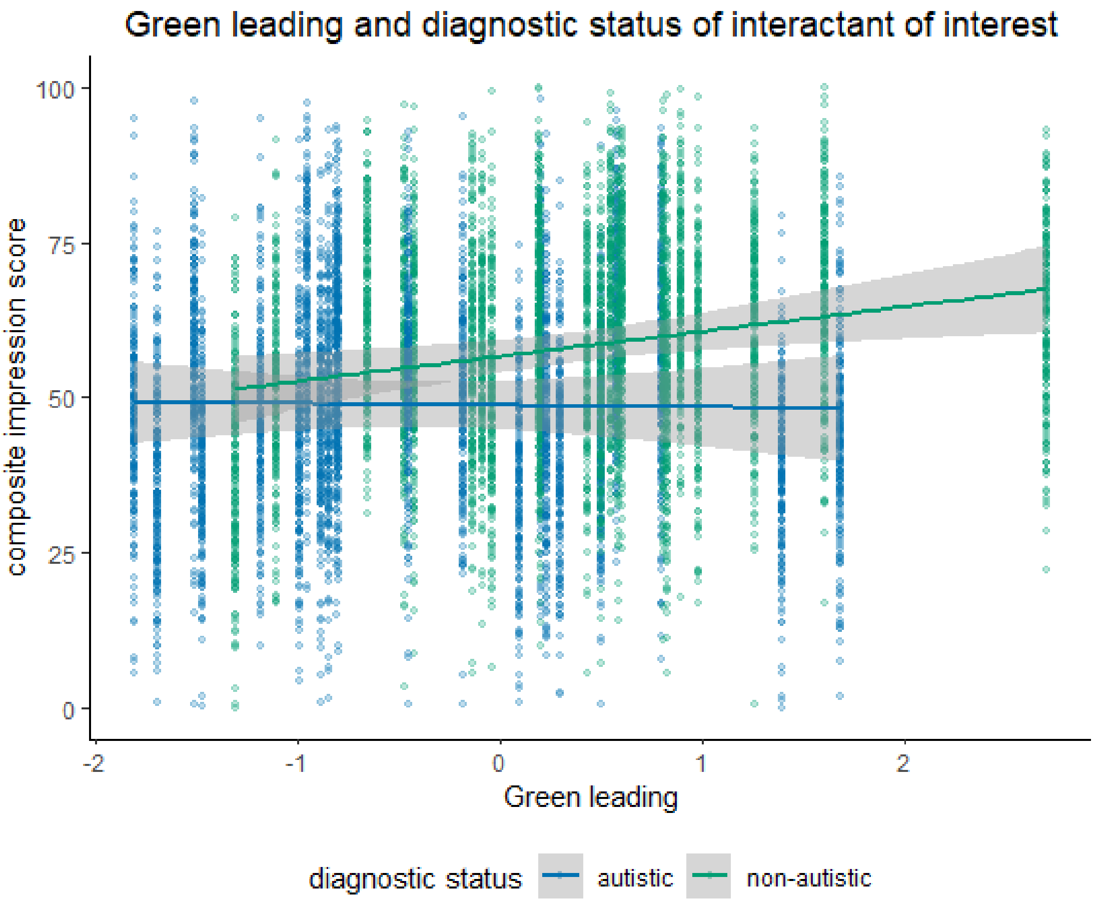

Figure 6: Association between impression and green interactant leading.

H4: fixations and diagnostic status of the interactant

Hypothesis 4 states that people fixate more often on interactants with autism due to idiosyncratic gestures associated with autism. We have the proportions of time that people looked at the left versus the right side of the screen. From this and the position of the interactants we can compute the percentage that people were watching the green autistic interactant and the green non-autistic interactant. We also need to exclude participants for whom the eye tracking did not work / worked badly. In the preprocessing of the eye tracking data, we have set a confidence threshold of 70% as well as a fixation threshold of 50ms. Only participants with at least 100 usable trials will be included in the analysis.

```
# first, we need to distinguish between the green (target) and the white
(non-target) person's side
df$green_prop = NA
```

```
df[df$position_true == "Links",]$green_prop  = df[df$position_true ==
"Links",]$L_prop
df[df$position_true == "Rechts",]$green_prop = df[df$position_true ==
"Rechts",]$R_prop

df$white_prop = NA
df[df$position_true == "Links",]$white_prop  = df[df$position_true ==
"Links",]$R_prop
df[df$position_true == "Rechts",]$white_prop = df[df$position_true ==
"Rechts",]$L_prop

# then, we create a new data frame, filtering out trials with less than 400
samples,
# and proportions as well as switches that are outliers according to the IQR
method.
# lastly, participants with less than 50% of the trials left are excluded.
df.et = df %>% mutate(
    et = Trial_number >= 400
  ) %>%
  replace_na(list(et = FALSE)) %>%
  filter(aq_high == F, et == T) %>%
  group_by(sub_id) %>%
  mutate(
    Vid_trials = n()
  ) %>% filter(Vid_trials >= 22)

# have a look at the remaining sample
df.et.sub = df.et %>% select(Alter, Geschlecht, Vid_trials) %>% distinct()

## Adding missing grouping variables: `sub_id`

summary(df.et.sub)

##       sub_id        Alter           Geschlecht    Vid_trials
##  5339563: 1   Min.   :19.00   männlich:30   Min.   :24.0
##  5416927: 1   1st Qu.:22.00   weiblich:61   1st Qu.:37.0
##  5419762: 1   Median :24.00                 Median :43.0
##  5420374: 1   Mean   :25.62                 Mean   :40.2
##  5428557: 1   3rd Qu.:27.00                 3rd Qu.:44.0
##  5432166: 1   Max.   :59.00                 Max.   :44.0
##  (Other):85

# let's do a sanity check: did they look more often at the green person?
df.san = df.et %>%
  select(sub, stm, white_prop, green_prop) %>%
  pivot_longer(cols = c(green_prop, white_prop), names_to = "target",
values_to = "proportion") %>%
  separate(target, c("target", "lose")) %>%
  select(-lose) %>% group_by(sub, target) %>%
  summarise(
```

```
    prop_agg = mean(proportion)
  ) %>% ungroup() %>%
  mutate(
    IQR   = IQR(prop_agg),
    upper = quantile(prop_agg, probs=c(0.75), na.rm = T) + 1.5*IQR,
    lower = quantile(prop_agg, probs=c(0.25), na.rm = T) - 1.5*IQR
  ) %>%
  filter(lower <= prop_agg & prop_agg <= upper)

## Adding missing grouping variables: `sub_id`
## `summarise()` has grouped output by 'sub'. You can override using the
`.groups`
## argument.

mean(df.san[df.san$target == "green",]$prop_agg)

## [1] 0.6447416

mean(df.san[df.san$target == "white",]$prop_agg)

## [1] 0.3552584

# assumptions
var.test(df.san[df.san$target == "white",]$prop_agg, df.san[df.san$target ==
"green",]$prop_agg)

##
##  F test to compare two variances
##
## data:  df.san[df.san$target == "white", ]$prop_agg and
df.san[df.san$target == "green", ]$prop_agg
## F = 1, num df = 90, denom df = 90, p-value = 1
## alternative hypothesis: true ratio of variances is not equal to 1
## 95 percent confidence interval:
##  0.6597954 1.5156213
## sample estimates:
## ratio of variances
##                  1

shapiro.test(df.san[df.san$target == "white",]$prop_agg)

##
##  Shapiro-Wilk normality test
##
## data:  df.san[df.san$target == "white", ]$prop_agg
## W = 0.98326, p-value = 0.2941

shapiro.test(df.san[df.san$target == "green",]$prop_agg)

##
##  Shapiro-Wilk normality test
##
```

```
## data:  df.san[df.san$target == "green", ]$prop_agg
## W = 0.98326, p-value = 0.2941

# Bayesian t-test
ttestBF(x = df.san[df.san$target == "white",]$prop_agg,
        y = df.san[df.san$target == "green",]$prop_agg,
        paired = T)

## Bayes factor analysis
## --------------
## [1] Alt., r=0.707 : 1.185461e+21 ±0%
##
## Against denominator:
##   Null, mu = 0
## ---
## Bayes factor type: BFoneSample, JZS

# plot the two
ggplot(df.san, aes(x = prop_agg, fill = target)) + geom_density(alpha=.3) +
  scale_fill_manual(values = c(gr,db))
```

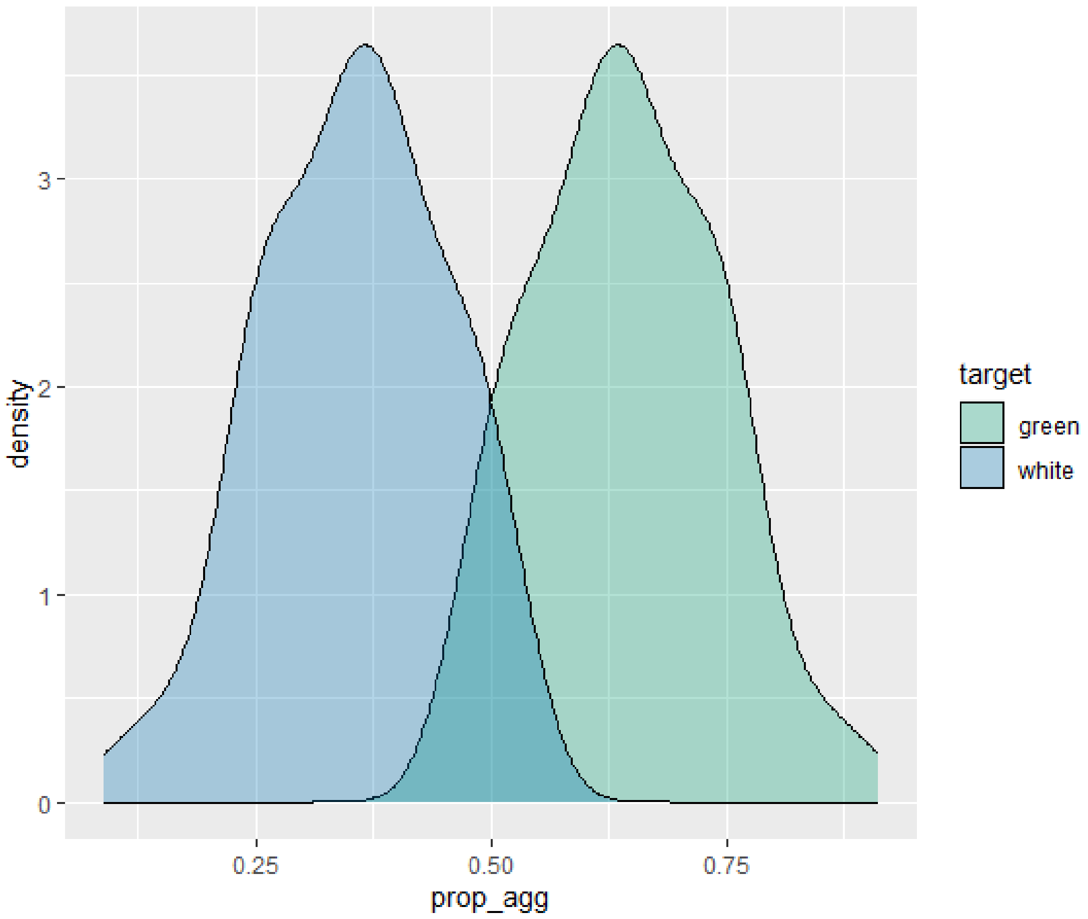

Figure 7: Proportion of the fixation time spent on the side of the green and the white person, showing that participants attending more strongly to the green interactant of interest.

Our sanity check works out nicely, showing that indeed the participants spent more time looking on the screen half that showed the green compared to the white person.

```
# let's check whether people looked more at the green person when it was an
autistic person?
df.prop = df.et %>%
  select(sub, stm, `diagnostic status`, green_prop) %>%
  group_by(sub, `diagnostic status`) %>%
  summarise(
    prop_agg = mean(green_prop)
  ) %>% ungroup() %>%
  mutate(
    IQR   = IQR(prop_agg),
    upper = quantile(prop_agg, probs=c(0.75), na.rm = T) + 1.5*IQR,
    lower = quantile(prop_agg, probs=c(0.25), na.rm = T) - 1.5*IQR
  ) %>%
  filter(lower <= prop_agg & prop_agg <= upper) %>%
  pivot_wider(names_from = `diagnostic status`, values_from = prop_agg) %>%
  drop_na()

## Adding missing grouping variables: `sub_id`
## `summarise()` has grouped output by 'sub'. You can override using the
`.groups`
## argument.

mean(df.prop$autistic)

## [1] 0.6500534

sd(df.prop$autistic)

## [1] 0.1042532

mean(df.prop$"non-autistic")

## [1] 0.6399938

sd(df.prop$"non-autistic")

## [1] 0.1049138

# assumptions
var.test(df.prop$autistic, df.prop$`non-autistic`)

##
##  F test to compare two variances
##
## data:  df.prop$autistic and df.prop$`non-autistic`
## F = 0.98745, num df = 90, denom df = 90, p-value = 0.9523
## alternative hypothesis: true ratio of variances is not equal to 1
## 95 percent confidence interval:
##  0.6515119 1.4965930
```

```
## sample estimates:
## ratio of variances
##          0.9874452
```

```
shapiro.test(df.prop$autistic)
```

```
##
##  Shapiro-Wilk normality test
##
## data:  df.prop$autistic
## W = 0.98451, p-value = 0.356
```

```
shapiro.test(df.prop$`non-autistic`)
```

```
##
##  Shapiro-Wilk normality test
##
## data:  df.prop$`non-autistic`
## W = 0.98284, p-value = 0.2755
```

```
# Bayesian t-test
ttestBF(x = df.prop$autistic,
        y = df.prop$`non-autistic`,
        paired = T)
```

```
## Bayes factor analysis
## --------------
## [1] Alt., r=0.707 : 0.2937007 ±0.06%
##
## Against denominator:
##   Null, mu = 0
## ---
## Bayes factor type: BFoneSample, JZS
```

```
# plot the two
ggplot(df.prop %>% pivot_longer(cols = c(autistic, `non-autistic`), names_to
= "diagnostic status"), aes(x = value, fill = `diagnostic status`)) +
geom_density(alpha=.3) +
  scale_fill_manual(values = c(gr,db))
```

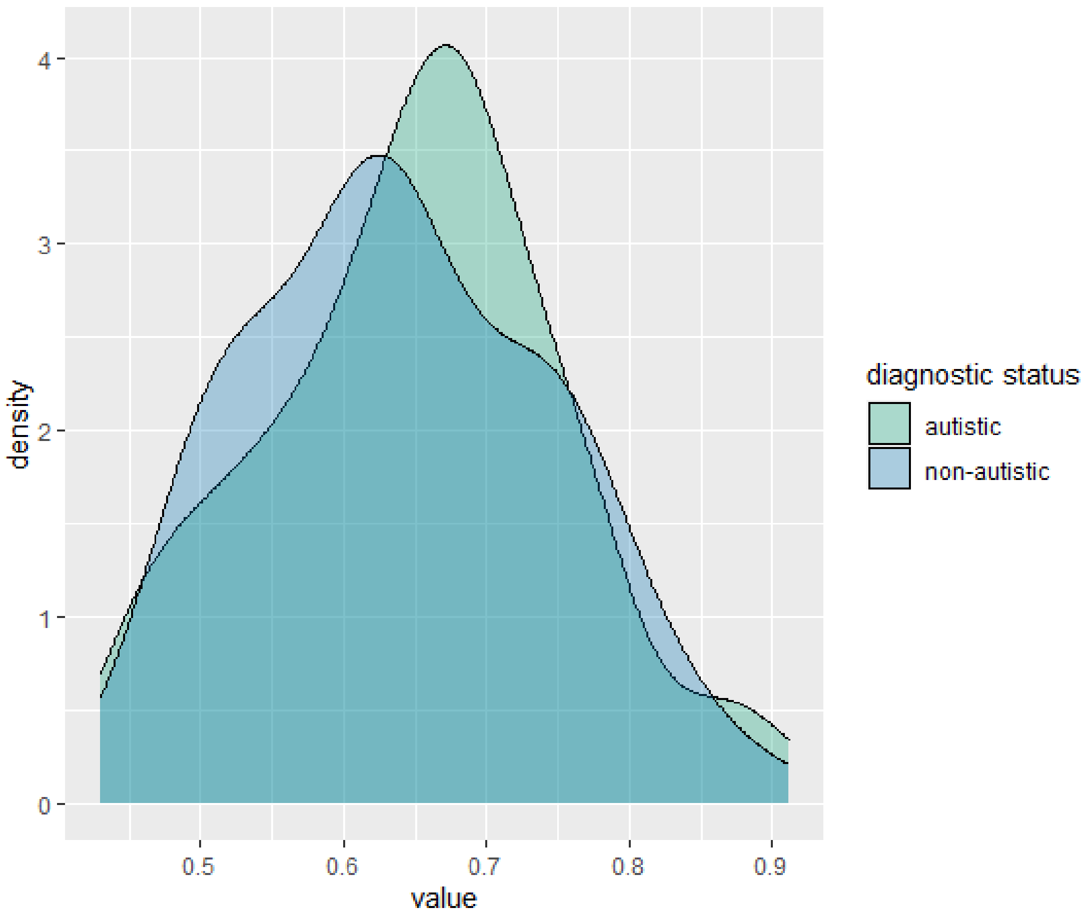

Figure 8: Proportion of time spent on autistic and non-autistic interactants of interest.

```
# let's check whether people performed more switches in the mixed condition?
df.switch = df.et %>%
  select(sub, stm, `diagnostic status`, Switches, Trial_number) %>%
  group_by(sub, `diagnostic status`) %>%
  summarise(
    switch_agg = (mean(Switches) * 100) / mean(Trial_number)
  ) %>% ungroup() %>%
  mutate(
    IQR   = IQR(switch_agg),
    upper = quantile(switch_agg, probs=c(0.75), na.rm = T) + 1.5*IQR,
    lower = quantile(switch_agg, probs=c(0.25), na.rm = T) - 1.5*IQR
  ) %>%
  filter(lower <= switch_agg & switch_agg <= upper) %>%
  pivot_wider(names_from = `diagnostic status`, values_from = switch_agg) %>%
  drop_na()

## Adding missing grouping variables: `sub_id`
## `summarise()` has grouped output by 'sub'. You can override using the
`.groups`
## argument.
```

```
mean(df.switch$autistic)

## [1] 5.548391

sd(df.switch$autistic)

## [1] 1.730978

mean(df.switch$"non-autistic")

## [1] 5.486091

sd(df.switch$"non-autistic")

## [1] 1.714835

# assumptions
var.test(df.switch$autistic, df.switch$`non-autistic`)

##
##  F test to compare two variances
##
## data:  df.switch$autistic and df.switch$`non-autistic`
## F = 1.0189, num df = 85, denom df = 85, p-value = 0.9314
## alternative hypothesis: true ratio of variances is not equal to 1
## 95 percent confidence interval:
##  0.6641145 1.5632713
## sample estimates:
## ratio of variances
##           1.018917

shapiro.test(df.switch$autistic)

##
##  Shapiro-Wilk normality test
##
## data:  df.switch$autistic
## W = 0.98225, p-value = 0.2858

shapiro.test(df.switch$`non-autistic`)

##
##  Shapiro-Wilk normality test
##
## data:  df.switch$`non-autistic`
## W = 0.9739, p-value = 0.07877

# Bayesian t-test
ttestBF(x = df.switch$autistic,
        y = df.switch$`non-autistic`,
        paired = T)
```

```
## Bayes factor analysis
## --------------
## [1] Alt., r=0.707 : 0.1561716 ±0.1%
##
## Against denominator:
##   Null, mu = 0
## ---
## Bayes factor type: BFoneSample, JZS

# plot the two
ggplot(df.switch %>% pivot_longer(cols = c(autistic, `non-autistic`),
names_to = "diagnostic status"), aes(x = value, fill = `diagnostic status`))
+ geom_density(alpha=.3)+
  scale_fill_manual(values = c(gr,db))
```

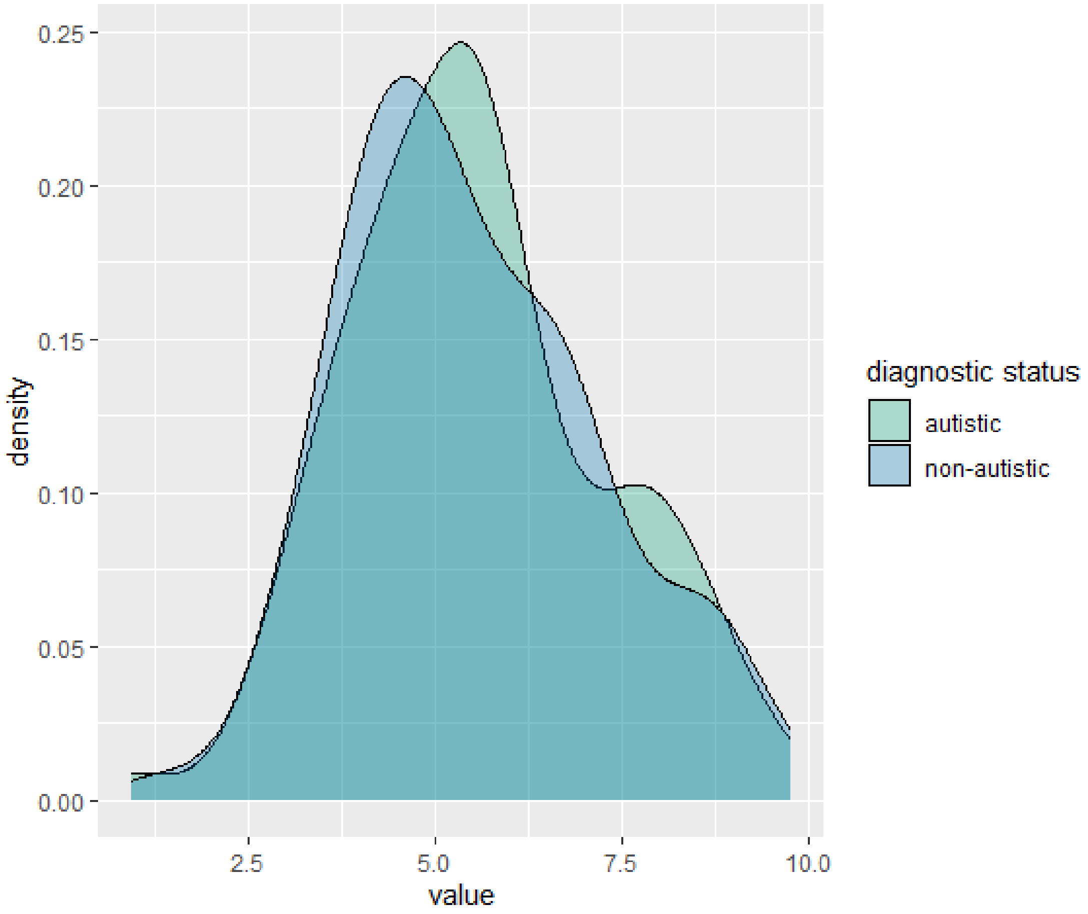

Figure 9: Distribution of switches per trial for videos with autistic and non-autistic interactants of interest.

Explorative analysis: people with high AQ values

Let's take a look at what happens if we don't exclude all people with an AQ score above 5, but rather compare them to the participants with lower AQ values. Will these two groups differ from each other?

```
if (file.exists("models/brm_imp-aq.rds")) {
  mimpaq = readRDS('models/brm_imp-aq.rds')
} else {
  mimpaq = brm(formula = imp ~
                 aq_high * `diagnostic status`* green_lead * white_lead + #
fixed effects
                 green_mot_head + white_mot_head + place + # regressors of no
interest
                 (`diagnostic status`+ place|sub) + (1|stm), # random effects
               data = df,
               control = list(adapt_delta = 0.99,
                              max_treedepth = 13),
               save_pars = save_pars(all = T),
               chains = 4,
               warmup = nw, iter = nw*2)
  saveRDS(mimpaq, file = 'models/brm_imp-aq.rds')
}

# check assumptions for this model
check_model(mimpaq,
check=c("qq","linearity","homogeneity","reqq","vif","pp_check"))
```

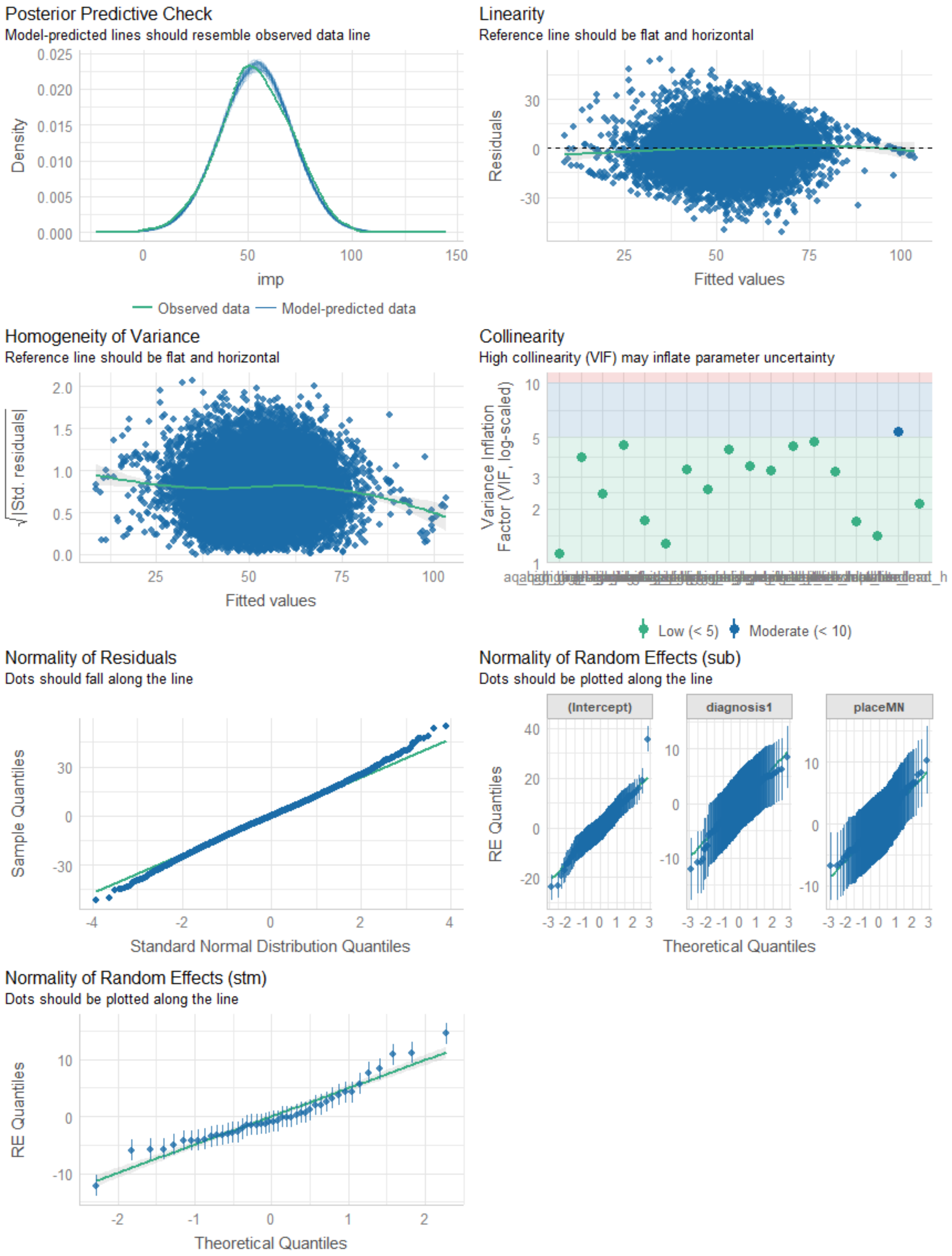

Figure 10: Model assumption check for the model including participants with high autism-like traits compared to participants with low-autistic participants.

```
# are there differences connected to the aq_high groups?
hypothesis(mimpaq, "aq_highTRUE > 0", alpha = 0.025)
```

```
## Hypothesis Tests for class b:
##          Hypothesis Estimate Est.Error CI.Lower CI.Upper Evid.Ratio
Post.Prob
## 1 (aq_highTRUE) > 0     0.83      1.32    -1.77      3.4       2.79
0.74
##   Star
## 1
## ---
## 'CI': 95%-CI for one-sided and 97.5%-CI for two-sided hypotheses.
## '*': For one-sided hypotheses, the posterior probability exceeds 97.5%;
## for two-sided hypotheses, the value tested against lies outside the 97.5%-
CI.
## Posterior probabilities of point hypotheses assume equal prior
probabilities.
```

```
hypothesis(mimpaq, "diagnosis1:aq_highTRUE > 0", alpha = 0.025)
```

```
## Hypothesis Tests for class b:
##                Hypothesis Estimate Est.Error CI.Lower CI.Upper Evid.Ratio
## 1 (diagnosis1:aq_hi... > 0     1.81      1.01    -0.14     3.79      27.94
##   Post.Prob Star
## 1      0.97
## ---
## 'CI': 95%-CI for one-sided and 97.5%-CI for two-sided hypotheses.
## '*': For one-sided hypotheses, the posterior probability exceeds 97.5%;
## for two-sided hypotheses, the value tested against lies outside the 97.5%-
CI.
## Posterior probabilities of point hypotheses assume equal prior
probabilities.
```

```
# get statistics
kable(df %>% group_by(aq_high, `diagnostic status`) %>%
  summarise(
    imp_avg = mean(imp, na.rm = T),
    imp_sd = sd(imp, na.rm = T)
  ))
```

```
## `summarise()` has grouped output by 'aq_high'. You can override using the
## `.groups` argument.
```

Table 2: Means in impression scores for participants with high and low autism-like traits for autistic and non-autistic interactants of interest.

aq_high	diagnostic status	imp_avg	imp_sd
FALSE	autistic	48.86105	17.17859
FALSE	non-autistic	58.10243	16.47337
TRUE	autistic	51.18429	15.93985
TRUE	non-autistic	58.96336	15.04769

There might be something connected to the interaction with the diagnostic status of the interactant. This could be worth checking out in a future study.

Explorative: separate ratings

Awkwardness

```
if (file.exists("models/brm_awk.rds")) {
  mawk = readRDS('models/brm_awk.rds')
} else {
  mawk = brm(formula = awk ~
               `diagnostic status`* green_lead * white_lead + # fixed effects
               green_mot_head + white_mot_head + place + # regressors of no
interest
                (`diagnostic status`+ place|sub) + (1|stm), # random effects
             data = df[df$aq_high == F,],
             control = list(adapt_delta = 0.99,
                            max_treedepth = 13),
             save_pars = save_pars(all = T),
             chains = 4,
             warmup = nw, iter = nw*2)
  saveRDS(mawk, file = 'models/brm_awk.rds')
}

# H1
hypothesis(mawk, "diagnosis1 < 0", alpha = 0.025)

## Hypothesis Tests for class b:
##          Hypothesis Estimate Est.Error CI.Lower CI.Upper Evid.Ratio
Post.Prob
## 1 (diagnosis1) < 0   -12.51       4.8   -22.05       -3     172.91
0.99
##   Star
## 1    *
## ---
## 'CI': 95%-CI for one-sided and 97.5%-CI for two-sided hypotheses.
## '*': For one-sided hypotheses, the posterior probability exceeds 97.5%;
## for two-sided hypotheses, the value tested against lies outside the 97.5%-
CI.
## Posterior probabilities of point hypotheses assume equal prior
probabilities.

# H2
hypothesis(mawk, "green_lead > 0", alpha = 0.025)

## Hypothesis Tests for class b:
##          Hypothesis Estimate Est.Error CI.Lower CI.Upper Evid.Ratio
Post.Prob
## 1 (green_lead) > 0     2.07      3.99    -5.75     9.85       2.33
0.7
```

```
##   Star
## 1
## ---
## 'CI': 95%-CI for one-sided and 97.5%-CI for two-sided hypotheses.
## '*': For one-sided hypotheses, the posterior probability exceeds 97.5%;
## for two-sided hypotheses, the value tested against lies outside the 97.5%-
CI.
## Posterior probabilities of point hypotheses assume equal prior
probabilities.
```

```
hypothesis(mawk, "white_lead > 0", alpha = 0.025)
```

```
## Hypothesis Tests for class b:
##         Hypothesis Estimate Est.Error CI.Lower CI.Upper Evid.Ratio
Post.Prob
## 1 (white_lead) > 0     0.82      4.28    -7.67     9.27       1.38
0.58
##   Star
## 1
## ---
## 'CI': 95%-CI for one-sided and 97.5%-CI for two-sided hypotheses.
## '*': For one-sided hypotheses, the posterior probability exceeds 97.5%;
## for two-sided hypotheses, the value tested against lies outside the 97.5%-
CI.
## Posterior probabilities of point hypotheses assume equal prior
probabilities.
```

```
# H3
hypothesis(mawk, "diagnosis1:green_lead < 0", alpha = 0.025)
```

```
## Hypothesis Tests for class b:
##                 Hypothesis Estimate Est.Error CI.Lower CI.Upper Evid.Ratio
## 1 (diagnosis1:green... < 0    -8.14      4.72   -17.46     1.16       22.5
##   Post.Prob Star
## 1      0.96
## ---
## 'CI': 95%-CI for one-sided and 97.5%-CI for two-sided hypotheses.
## '*': For one-sided hypotheses, the posterior probability exceeds 97.5%;
## for two-sided hypotheses, the value tested against lies outside the 97.5%-
CI.
## Posterior probabilities of point hypotheses assume equal prior
probabilities.
```

```
hypothesis(mawk, "diagnosis1:white_lead > 0", alpha = 0.025)
```

```
## Hypothesis Tests for class b:
##                 Hypothesis Estimate Est.Error CI.Lower CI.Upper Evid.Ratio
## 1 (diagnosis1:white... > 0     1.55      4.82    -7.88    11.17       1.68
##   Post.Prob Star
## 1      0.63
## ---
```

```
## 'CI': 95%-CI for one-sided and 97.5%-CI for two-sided hypotheses.
## '*': For one-sided hypotheses, the posterior probability exceeds 97.5%;
## for two-sided hypotheses, the value tested against lies outside the 97.5%-
CI.
## Posterior probabilities of point hypotheses assume equal prior
probabilities.

hypothesis(mawk, "diagnosis1:green_lead:white_lead > 0", alpha = 0.025)

## Hypothesis Tests for class b:
##                Hypothesis Estimate Est.Error CI.Lower CI.Upper Evid.Ratio
## 1 (diagnosis1:green... > 0     3.13      4.09    -4.92    11.18       3.63
##   Post.Prob Star
## 1      0.78
## ---
## 'CI': 95%-CI for one-sided and 97.5%-CI for two-sided hypotheses.
## '*': For one-sided hypotheses, the posterior probability exceeds 97.5%;
## for two-sided hypotheses, the value tested against lies outside the 97.5%-
CI.
## Posterior probabilities of point hypotheses assume equal prior
probabilities.
```

Friends

```
if (file.exists("models/brm_fri.rds")) {
  mfri = readRDS('models/brm_fri.rds')
} else {
  mfri = brm(formula = fri ~
               `diagnostic status`* green_lead * white_lead + # fixed effects
               green_mot_head + white_mot_head + place + # regressors of no
interest
               (`diagnostic status`+ place|sub) + (1|stm), # random effects
             data = df[df$aq_high == F,],
             control = list(adapt_delta = 0.99,
                            max_treedepth = 13),
             save_pars = save_pars(all = T),
             chains = 4,
             warmup = nw, iter = nw*2)
  saveRDS(mfri, file = 'models/brm_fri.rds')
}

# H1
hypothesis(mfri, "diagnosis1 < 0", alpha = 0.025)

## Hypothesis Tests for class b:
##         Hypothesis Estimate Est.Error CI.Lower CI.Upper Evid.Ratio
Post.Prob
## 1 (diagnosis1) < 0    -7.51      3.66   -14.67    -0.22      43.94
0.98
##   Star
## 1    *
```

```
## ---
## 'CI': 95%-CI for one-sided and 97.5%-CI for two-sided hypotheses.
## '*': For one-sided hypotheses, the posterior probability exceeds 97.5%;
## for two-sided hypotheses, the value tested against lies outside the 97.5%-
CI.
## Posterior probabilities of point hypotheses assume equal prior
probabilities.

# H2
hypothesis(mfri, "green_lead > 0", alpha = 0.025)

## Hypothesis Tests for class b:
##          Hypothesis Estimate Est.Error CI.Lower CI.Upper Evid.Ratio
Post.Prob
## 1 (green_lead) > 0     2.46      3.07    -3.67     8.51       3.88
0.8
##   Star
## 1
## ---
## 'CI': 95%-CI for one-sided and 97.5%-CI for two-sided hypotheses.
## '*': For one-sided hypotheses, the posterior probability exceeds 97.5%;
## for two-sided hypotheses, the value tested against lies outside the 97.5%-
CI.
## Posterior probabilities of point hypotheses assume equal prior
probabilities.

hypothesis(mfri, "white_lead < 0", alpha = 0.025)

## Hypothesis Tests for class b:
##          Hypothesis Estimate Est.Error CI.Lower CI.Upper Evid.Ratio
Post.Prob
## 1 (white_lead) < 0    -0.56      3.31    -7.05     6.05       1.33
0.57
##   Star
## 1
## ---
## 'CI': 95%-CI for one-sided and 97.5%-CI for two-sided hypotheses.
## '*': For one-sided hypotheses, the posterior probability exceeds 97.5%;
## for two-sided hypotheses, the value tested against lies outside the 97.5%-
CI.
## Posterior probabilities of point hypotheses assume equal prior
probabilities.

# H3
hypothesis(mfri, "diagnosis1:green_lead < 0", alpha = 0.025)

## Hypothesis Tests for class b:
##                  Hypothesis Estimate Est.Error CI.Lower CI.Upper Evid.Ratio
## 1 (diagnosis1:green... < 0    -5.92      3.67    -13.1     1.35      17.38
##   Post.Prob Star
## 1      0.95
```

```
## ---
## 'CI': 95%-CI for one-sided and 97.5%-CI for two-sided hypotheses.
## '*': For one-sided hypotheses, the posterior probability exceeds 97.5%;
## for two-sided hypotheses, the value tested against lies outside the 97.5%-
CI.
## Posterior probabilities of point hypotheses assume equal prior
probabilities.
```

```
hypothesis(mfri, "diagnosis1:white_lead > 0", alpha = 0.025)
```

```
## Hypothesis Tests for class b:
##                 Hypothesis Estimate Est.Error CI.Lower CI.Upper Evid.Ratio
## 1 (diagnosis1:white... > 0     0.21      3.75    -7.21     7.59        1.1
##   Post.Prob Star
## 1      0.52
## ---
## 'CI': 95%-CI for one-sided and 97.5%-CI for two-sided hypotheses.
## '*': For one-sided hypotheses, the posterior probability exceeds 97.5%;
## for two-sided hypotheses, the value tested against lies outside the 97.5%-
CI.
## Posterior probabilities of point hypotheses assume equal prior
probabilities.
```

```
hypothesis(mfri, "diagnosis1:green_lead:white_lead < 0", alpha = 0.025)
```

```
## Hypothesis Tests for class b:
##                 Hypothesis Estimate Est.Error CI.Lower CI.Upper Evid.Ratio
## 1 (diagnosis1:green... < 0    -1.44      3.12     -7.7     4.67       2.13
##   Post.Prob Star
## 1      0.68
## ---
## 'CI': 95%-CI for one-sided and 97.5%-CI for two-sided hypotheses.
## '*': For one-sided hypotheses, the posterior probability exceeds 97.5%;
## for two-sided hypotheses, the value tested against lies outside the 97.5%-
CI.
## Posterior probabilities of point hypotheses assume equal prior
probabilities.
```

Intelligence

```
if (file.exists("models/brm_int.rds")) {
  mint = readRDS('models/brm_int.rds')
} else {
  mint = brm(formula = int ~
               `diagnostic status`* green_lead * white_lead + # fixed effects
               green_mot_head + white_mot_head + place + # regressors of no
interest
                (`diagnostic status`+ place|sub) + (1|stm), # random effects
             data = df[df$aq_high == F,],
             control = list(adapt_delta = 0.99,
                            max_treedepth = 13),
             save_pars = save_pars(all = T),
```

```
             chains = 4,
             warmup = nw, iter = nw*2)
  saveRDS(mint, file = 'models/brm_int.rds')
}

# H1
hypothesis(mint, "diagnosis1 < 0", alpha = 0.025)

## Hypothesis Tests for class b:
##          Hypothesis Estimate Est.Error CI.Lower CI.Upper Evid.Ratio
Post.Prob
## 1 (diagnosis1) < 0    -1.96      2.66    -7.18     3.28       3.39
0.77
##   Star
## 1
## ---
## 'CI': 95%-CI for one-sided and 97.5%-CI for two-sided hypotheses.
## '*': For one-sided hypotheses, the posterior probability exceeds 97.5%;
## for two-sided hypotheses, the value tested against lies outside the 97.5%-
CI.
## Posterior probabilities of point hypotheses assume equal prior
probabilities.

# H2
hypothesis(mint, "green_lead > 0", alpha = 0.025)

## Hypothesis Tests for class b:
##          Hypothesis Estimate Est.Error CI.Lower CI.Upper Evid.Ratio
Post.Prob
## 1 (green_lead) > 0     6.03      2.21     1.71    10.39     226.27
1
##   Star
## 1    *
## ---
## 'CI': 95%-CI for one-sided and 97.5%-CI for two-sided hypotheses.
## '*': For one-sided hypotheses, the posterior probability exceeds 97.5%;
## for two-sided hypotheses, the value tested against lies outside the 97.5%-
CI.
## Posterior probabilities of point hypotheses assume equal prior
probabilities.

hypothesis(mint, "white_lead < 0", alpha = 0.025)

## Hypothesis Tests for class b:
##          Hypothesis Estimate Est.Error CI.Lower CI.Upper Evid.Ratio
Post.Prob
## 1 (white_lead) < 0    -2.71      2.38    -7.38     2.01       7.05
0.88
##   Star
## 1
## ---
```

```
## 'CI': 95%-CI for one-sided and 97.5%-CI for two-sided hypotheses.
## '*': For one-sided hypotheses, the posterior probability exceeds 97.5%;
## for two-sided hypotheses, the value tested against lies outside the 97.5%-
CI.
## Posterior probabilities of point hypotheses assume equal prior
probabilities.
```

```
# H3
hypothesis(mint, "diagnosis1:green_lead < 0", alpha = 0.025)
```

```
## Hypothesis Tests for class b:
##                Hypothesis Estimate Est.Error CI.Lower CI.Upper Evid.Ratio
## 1 (diagnosis1:green... < 0    -7.94      2.68   -13.31    -2.66     688.66
##   Post.Prob Star
## 1         1    *
## ---
## 'CI': 95%-CI for one-sided and 97.5%-CI for two-sided hypotheses.
## '*': For one-sided hypotheses, the posterior probability exceeds 97.5%;
## for two-sided hypotheses, the value tested against lies outside the 97.5%-
CI.
## Posterior probabilities of point hypotheses assume equal prior
probabilities.
```

```
hypothesis(mint, "diagnosis1:white_lead > 0", alpha = 0.025)
```

```
## Hypothesis Tests for class b:
##                Hypothesis Estimate Est.Error CI.Lower CI.Upper Evid.Ratio
## 1 (diagnosis1:white... > 0     2.68      2.71    -2.65        8       5.42
##   Post.Prob Star
## 1      0.84
## ---
## 'CI': 95%-CI for one-sided and 97.5%-CI for two-sided hypotheses.
## '*': For one-sided hypotheses, the posterior probability exceeds 97.5%;
## for two-sided hypotheses, the value tested against lies outside the 97.5%-
CI.
## Posterior probabilities of point hypotheses assume equal prior
probabilities.
```

```
hypothesis(mint, "diagnosis1:green_lead:white_lead > 0", alpha = 0.025)
```

```
## Hypothesis Tests for class b:
##                Hypothesis Estimate Est.Error CI.Lower CI.Upper Evid.Ratio
## 1 (diagnosis1:green... > 0     0.96       2.3    -3.58      5.5       1.99
##   Post.Prob Star
## 1      0.67
## ---
## 'CI': 95%-CI for one-sided and 97.5%-CI for two-sided hypotheses.
## '*': For one-sided hypotheses, the posterior probability exceeds 97.5%;
## for two-sided hypotheses, the value tested against lies outside the 97.5%-
CI.
```

```
## Posterior probabilities of point hypotheses assume equal prior
probabilities.
```

Likeability

```
if (file.exists("models/brm_lik.rds")) {
  mlik = readRDS('models/brm_lik.rds')
} else {
  mlik = brm(formula = lik ~
               `diagnostic status`* green_lead * white_lead + # fixed effects
               green_mot_head + white_mot_head + place + # regressors of no
interest
                (`diagnostic status`+ place|sub) + (1|stm), # random effects
             data = df[df$aq_high == F,],
             control = list(adapt_delta = 0.99,
                            max_treedepth = 13),
             save_pars = save_pars(all = T),
             chains = 4,
             warmup = nw, iter = nw*2)
  saveRDS(mlik, file = 'models/brm_lik.rds')
}

# H1
hypothesis(mlik, "diagnosis1 < 0", alpha = 0.025)

## Hypothesis Tests for class b:
##          Hypothesis Estimate Est.Error CI.Lower CI.Upper Evid.Ratio
Post.Prob
## 1 (diagnosis1) < 0    -2.33      2.55    -7.32     2.71       4.76
0.83
##   Star
## 1
## ---
## 'CI': 95%-CI for one-sided and 97.5%-CI for two-sided hypotheses.
## '*': For one-sided hypotheses, the posterior probability exceeds 97.5%;
## for two-sided hypotheses, the value tested against lies outside the 97.5%-
CI.
## Posterior probabilities of point hypotheses assume equal prior
probabilities.

# H2
hypothesis(mlik, "green_lead > 0", alpha = 0.025)

## Hypothesis Tests for class b:
##          Hypothesis Estimate Est.Error CI.Lower CI.Upper Evid.Ratio
Post.Prob
## 1 (green_lead) > 0     5.08      2.09     0.96     9.18     115.96
0.99
##   Star
## 1    *
## ---
```

```
## 'CI': 95%-CI for one-sided and 97.5%-CI for two-sided hypotheses.
## '*': For one-sided hypotheses, the posterior probability exceeds 97.5%;
## for two-sided hypotheses, the value tested against lies outside the 97.5%-
CI.
## Posterior probabilities of point hypotheses assume equal prior
probabilities.
```

```
hypothesis(mlik, "white_lead < 0", alpha = 0.025)
```

```
## Hypothesis Tests for class b:
##         Hypothesis Estimate Est.Error CI.Lower CI.Upper Evid.Ratio
Post.Prob
## 1 (white_lead) < 0    -4.34      2.24    -8.73     0.05      37.31
0.97
##   Star
## 1
## ---
## 'CI': 95%-CI for one-sided and 97.5%-CI for two-sided hypotheses.
## '*': For one-sided hypotheses, the posterior probability exceeds 97.5%;
## for two-sided hypotheses, the value tested against lies outside the 97.5%-
CI.
## Posterior probabilities of point hypotheses assume equal prior
probabilities.
```

```
# H3
hypothesis(mlik, "diagnosis1:green_lead < 0", alpha = 0.025)
```

```
## Hypothesis Tests for class b:
##                Hypothesis Estimate Est.Error CI.Lower CI.Upper Evid.Ratio
## 1 (diagnosis1:green... < 0    -5.53       2.5   -10.44    -0.55      63.94
##   Post.Prob Star
## 1      0.98    *
## ---
## 'CI': 95%-CI for one-sided and 97.5%-CI for two-sided hypotheses.
## '*': For one-sided hypotheses, the posterior probability exceeds 97.5%;
## for two-sided hypotheses, the value tested against lies outside the 97.5%-
CI.
## Posterior probabilities of point hypotheses assume equal prior
probabilities.
```

```
hypothesis(mlik, "diagnosis1:white_lead > 0", alpha = 0.025)
```

```
## Hypothesis Tests for class b:
##                Hypothesis Estimate Est.Error CI.Lower CI.Upper Evid.Ratio
## 1 (diagnosis1:white... > 0     2.85      2.57    -2.24     7.84       6.62
##   Post.Prob Star
## 1      0.87
## ---
## 'CI': 95%-CI for one-sided and 97.5%-CI for two-sided hypotheses.
## '*': For one-sided hypotheses, the posterior probability exceeds 97.5%;
## for two-sided hypotheses, the value tested against lies outside the 97.5%-
```

```
CI.
## Posterior probabilities of point hypotheses assume equal prior
probabilities.
```

```
hypothesis(mlik, "diagnosis1:green_lead:white_lead < 0", alpha = 0.025)
```

```
## Hypothesis Tests for class b:
##                 Hypothesis Estimate Est.Error CI.Lower CI.Upper Evid.Ratio
## 1 (diagnosis1:green... < 0    -1.54      2.18    -5.82     2.69       3.19
##   Post.Prob Star
## 1      0.76
## ---
## 'CI': 95%-CI for one-sided and 97.5%-CI for two-sided hypotheses.
## '*': For one-sided hypotheses, the posterior probability exceeds 97.5%;
## for two-sided hypotheses, the value tested against lies outside the 97.5%-
CI.
## Posterior probabilities of point hypotheses assume equal prior
probabilities.
```

Talk

```
if (file.exists("models/brm_tlk.rds")) {
  mtlk = readRDS('models/brm_tlk.rds')
} else {
  mtlk = brm(formula = tlk ~
               `diagnostic status`* green_lead * white_lead + # fixed effects
               green_mot_head + white_mot_head + place + # regressors of no
interest
               (`diagnostic status`+ place|sub) + (1|stm), # random effects
             data = df[df$aq_high == F,],
             control = list(adapt_delta = 0.99,
                            max_treedepth = 13),
             save_pars = save_pars(all = T),
             chains = 4,
             warmup = nw, iter = nw*2)
  saveRDS(mtlk, file = 'models/brm_tlk.rds')
}

# H1
hypothesis(mtlk, "diagnosis1 < 0", alpha = 0.025)
```

```
## Hypothesis Tests for class b:
##       Hypothesis Estimate Est.Error CI.Lower CI.Upper Evid.Ratio
Post.Prob
## 1 (diagnosis1) < 0     -3.4      2.67    -8.61      1.8       9.08
0.9
##   Star
## 1
## ---
## 'CI': 95%-CI for one-sided and 97.5%-CI for two-sided hypotheses.
## '*': For one-sided hypotheses, the posterior probability exceeds 97.5%;
```

```
## for two-sided hypotheses, the value tested against lies outside the 97.5%-
CI.
## Posterior probabilities of point hypotheses assume equal prior
probabilities.

# H2
hypothesis(mtlk, "green_lead > 0", alpha = 0.025)

## Hypothesis Tests for class b:
##          Hypothesis Estimate Est.Error CI.Lower CI.Upper Evid.Ratio
Post.Prob
## 1 (green_lead) > 0     5.79      2.23     1.37    10.24     151.67
0.99
##   Star
## 1    *
## ---
## 'CI': 95%-CI for one-sided and 97.5%-CI for two-sided hypotheses.
## '*': For one-sided hypotheses, the posterior probability exceeds 97.5%;
## for two-sided hypotheses, the value tested against lies outside the 97.5%-
CI.
## Posterior probabilities of point hypotheses assume equal prior
probabilities.

hypothesis(mtlk, "white_lead < 0", alpha = 0.025)

## Hypothesis Tests for class b:
##          Hypothesis Estimate Est.Error CI.Lower CI.Upper Evid.Ratio
Post.Prob
## 1 (white_lead) < 0    -4.07      2.42    -8.84     0.78      20.37
0.95
##   Star
## 1
## ---
## 'CI': 95%-CI for one-sided and 97.5%-CI for two-sided hypotheses.
## '*': For one-sided hypotheses, the posterior probability exceeds 97.5%;
## for two-sided hypotheses, the value tested against lies outside the 97.5%-
CI.
## Posterior probabilities of point hypotheses assume equal prior
probabilities.

# H3
hypothesis(mtlk, "diagnosis1:green_lead < 0", alpha = 0.025)

## Hypothesis Tests for class b:
##                  Hypothesis Estimate Est.Error CI.Lower CI.Upper Evid.Ratio
## 1 (diagnosis1:green... < 0     -7.2       2.7   -12.47    -1.84     162.93
##   Post.Prob Star
## 1      0.99    *
## ---
## 'CI': 95%-CI for one-sided and 97.5%-CI for two-sided hypotheses.
## '*': For one-sided hypotheses, the posterior probability exceeds 97.5%;
```

```
## for two-sided hypotheses, the value tested against lies outside the 97.5%-
CI.
## Posterior probabilities of point hypotheses assume equal prior
probabilities.

hypothesis(mtlk, "diagnosis1:white_lead > 0", alpha = 0.025)

## Hypothesis Tests for class b:
##                 Hypothesis Estimate Est.Error CI.Lower CI.Upper Evid.Ratio
## 1 (diagnosis1:white... > 0     2.94      2.75    -2.55     8.26       6.26
##   Post.Prob Star
## 1      0.86
## ---
## 'CI': 95%-CI for one-sided and 97.5%-CI for two-sided hypotheses.
## '*': For one-sided hypotheses, the posterior probability exceeds 97.5%;
## for two-sided hypotheses, the value tested against lies outside the 97.5%-
CI.
## Posterior probabilities of point hypotheses assume equal prior
probabilities.

hypothesis(mtlk, "diagnosis1:green_lead:white_lead < 0", alpha = 0.025)

## Hypothesis Tests for class b:
##                 Hypothesis Estimate Est.Error CI.Lower CI.Upper Evid.Ratio
## 1 (diagnosis1:green... < 0    -0.91      2.29     -5.4     3.61       1.89
##   Post.Prob Star
## 1      0.65
## ---
## 'CI': 95%-CI for one-sided and 97.5%-CI for two-sided hypotheses.
## '*': For one-sided hypotheses, the posterior probability exceeds 97.5%;
## for two-sided hypotheses, the value tested against lies outside the 97.5%-
CI.
## Posterior probabilities of point hypotheses assume equal prior
probabilities.
```

Trustworthiness

```
if (file.exists("models/brm_tru.rds")) {
  mtru = readRDS('models/brm_tru.rds')
} else {
  mtru = brm(formula = tru ~
               `diagnostic status`* green_lead * white_lead + # fixed effects
               green_mot_head + white_mot_head + place + # regressors of no
interest
               (`diagnostic status`+ place|sub) + (1|stm), # random effects
             data = df[df$aq_high == F,],
             control = list(adapt_delta = 0.99,
                            max_treedepth = 13),
             save_pars = save_pars(all = T),
             chains = 4,
             warmup = nw, iter = nw*2)
  saveRDS(mtru, file = 'models/brm_tru.rds')
```

```
}

# H1
hypothesis(mtru, "diagnosis1 < 0", alpha = 0.025)

## Hypothesis Tests for class b:
##          Hypothesis Estimate Est.Error CI.Lower CI.Upper Evid.Ratio
Post.Prob
## 1 (diagnosis1) < 0    -2.26       2.5    -7.13     2.73       4.61
0.82
##   Star
## 1
## ---
## 'CI': 95%-CI for one-sided and 97.5%-CI for two-sided hypotheses.
## '*': For one-sided hypotheses, the posterior probability exceeds 97.5%;
## for two-sided hypotheses, the value tested against lies outside the 97.5%-
CI.
## Posterior probabilities of point hypotheses assume equal prior
probabilities.

# H2
hypothesis(mtru, "green_lead > 0", alpha = 0.025)

## Hypothesis Tests for class b:
##          Hypothesis Estimate Est.Error CI.Lower CI.Upper Evid.Ratio
Post.Prob
## 1 (green_lead) > 0     5.68      2.07     1.61     9.75     218.78
1
##   Star
## 1    *
## ---
## 'CI': 95%-CI for one-sided and 97.5%-CI for two-sided hypotheses.
## '*': For one-sided hypotheses, the posterior probability exceeds 97.5%;
## for two-sided hypotheses, the value tested against lies outside the 97.5%-
CI.
## Posterior probabilities of point hypotheses assume equal prior
probabilities.

hypothesis(mtru, "white_lead < 0", alpha = 0.025)

## Hypothesis Tests for class b:
##          Hypothesis Estimate Est.Error CI.Lower CI.Upper Evid.Ratio
Post.Prob
## 1 (white_lead) < 0    -4.92      2.19    -9.26    -0.64       81.3
0.99
##   Star
## 1    *
## ---
## 'CI': 95%-CI for one-sided and 97.5%-CI for two-sided hypotheses.
## '*': For one-sided hypotheses, the posterior probability exceeds 97.5%;
## for two-sided hypotheses, the value tested against lies outside the 97.5%-
```

```
CI.
## Posterior probabilities of point hypotheses assume equal prior
probabilities.

# H3
hypothesis(mtru, "diagnosis1:green_lead < 0", alpha = 0.025)

## Hypothesis Tests for class b:
##                Hypothesis Estimate Est.Error CI.Lower CI.Upper Evid.Ratio
## 1 (diagnosis1:green... < 0    -5.92      2.49   -10.88    -0.98     105.95
##   Post.Prob Star
## 1      0.99    *
## ---
## 'CI': 95%-CI for one-sided and 97.5%-CI for two-sided hypotheses.
## '*': For one-sided hypotheses, the posterior probability exceeds 97.5%;
## for two-sided hypotheses, the value tested against lies outside the 97.5%-
CI.
## Posterior probabilities of point hypotheses assume equal prior
probabilities.

hypothesis(mtru, "diagnosis1:white_lead > 0", alpha = 0.025)

## Hypothesis Tests for class b:
##                Hypothesis Estimate Est.Error CI.Lower CI.Upper Evid.Ratio
## 1 (diagnosis1:white... > 0     4.21      2.51    -0.72     9.21      21.05
##   Post.Prob Star
## 1      0.95
## ---
## 'CI': 95%-CI for one-sided and 97.5%-CI for two-sided hypotheses.
## '*': For one-sided hypotheses, the posterior probability exceeds 97.5%;
## for two-sided hypotheses, the value tested against lies outside the 97.5%-
CI.
## Posterior probabilities of point hypotheses assume equal prior
probabilities.

hypothesis(mtru, "diagnosis1:green_lead:white_lead < 0", alpha = 0.025)

## Hypothesis Tests for class b:
##                Hypothesis Estimate Est.Error CI.Lower CI.Upper Evid.Ratio
## 1 (diagnosis1:green... < 0    -0.58      2.15    -4.78     3.68       1.58
##   Post.Prob Star
## 1      0.61
## ---
## 'CI': 95%-CI for one-sided and 97.5%-CI for two-sided hypotheses.
## '*': For one-sided hypotheses, the posterior probability exceeds 97.5%;
## for two-sided hypotheses, the value tested against lies outside the 97.5%-
CI.
## Posterior probabilities of point hypotheses assume equal prior
probabilities.
```
